# Natural-derived acetophenones: chemistry and pharmacological activities

**DOI:** 10.1007/s13659-024-00447-x

**Published:** 2024-05-10

**Authors:** Hamid Ahmadpourmir, Homayoun Attar, Javad Asili, Vahid Soheili, Seyedeh Faezeh Taghizadeh, Abolfazl Shakeri

**Affiliations:** 1 Department of Pharmacognosy, School of Pharmacy, Mashhad University of Medical Sciences, Mashhad, Iran; 2 Department of Pharmaceutical Control, School of Pharmacy, Mashhad University of Medical Sciences, Mashhad, Iran

**Keywords:** Acetophenones, Bioactivity, *Melicope*, Phytochemicals

## Abstract

Acetophenones are naturally occurring phenolic compounds which have found in over 24 plant families and also fungi strains. They are exist in both free or glycosides form in nature. The biological activities of these compounds have been assayed and reported including cytotoxicity, antimicrobial, antimalarial, antioxidant and antityrosinase activities. Herein, we review the chemistry and biological activity of natural acetophenone derivatives that have been isolated and identified until January 2024. Taken together, it was reported 252 acetophenone derivatives in which the genera *Melicope* (69) and *Acronychia* (44) were the principal species as producers of acetophenones.

**Graphical Abstract:**

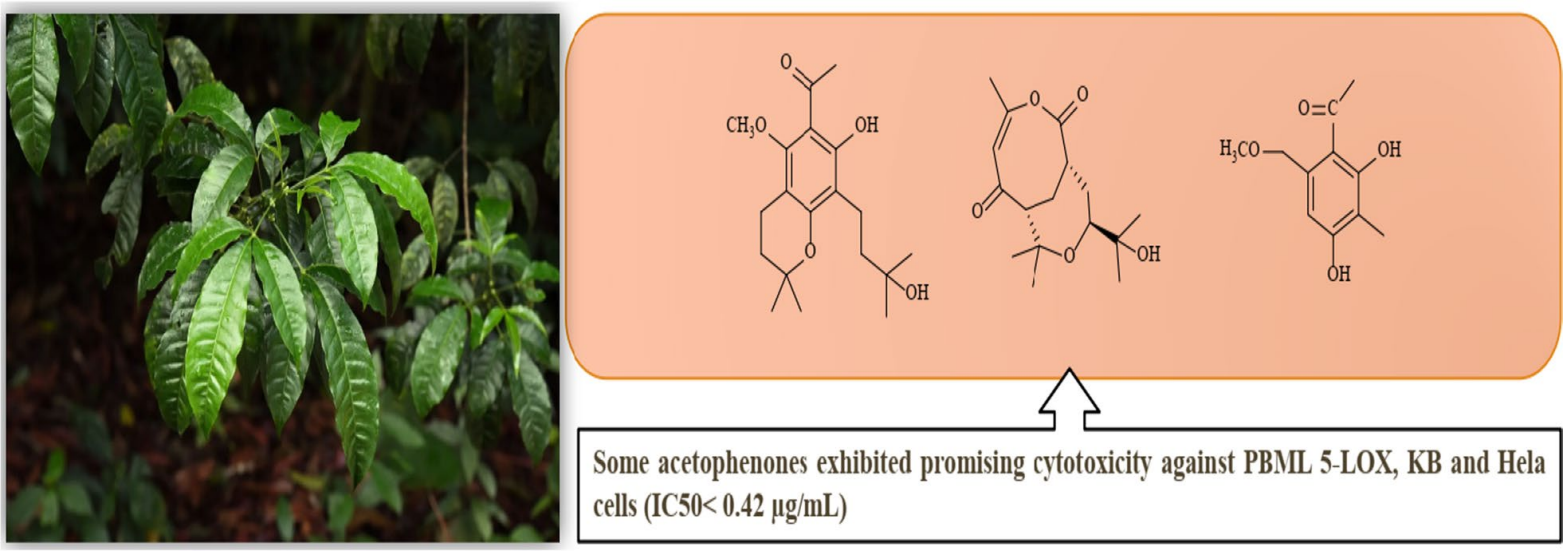

## Introduction

Acetophenones, as a group of phenolic compounds, produced by many plants of various families for some reasons such as repelling insects [1]. The proven ability of acetophenone-rich plants to fend off pests and insects has shed light on the perspective of using acetophenone derivatives as pesticides. With the crops suffering catastrophic losses due to pest attacks and diseases, and the public opinion bending towards mitigating the use of chemical pesticides, acetophenone rises as a candidate for an eco-friendly alternative for synthetic pesticides [2]. Plant-derived acetophenones, also, are important precursors for drug production. For example, acetophenone derivatives such as apocynin (**207**) and paeonol (**217**) show anti-inflammatory traits without any negative side effects, which make them perfect option for synthesizing drugs. The use of plants containing paeonol in folk medicine for their therapeutic properties dates back to a millennia ago [3]. Acetophenones can also contribute more to therapeutic applications due to their other biological activities such as anticancer, analgesic, antioxidant, cardioprotective, neuroprotective, and antidiabetic [4]. In addition to their biological applications, acetophenone derivatives are used in the food and fragrance industries, primarily for their orange blossom flavor [5]. They are also used as fragrance ingredients in detergents, soaps, and perfumes. In addition, acetophenones and their derivatives also have a variety of applications in the cosmetics production, especially in the making of odorless and colorless cosmetics with good antiseptic effects [6]. They are also used in the production of sunscreen products to protect against UV radiation [7]. Additionally, acetophenone compounds are employed in the production of alcohol, aldehydes, resins, esters, fragrances, and pharmaceuticals. They are important intermediates for the synthesis of natural products and marketed drugs, and they find wide use in fields such as biology, pesticides, polymers, and materials science. Moreover, they are ideal synthons for multicomponent reactions, including three- and four-component reactions, due to their commercial availability and accessibility [8]. Furthermore, highly functionalized acetophenone derivatives have interesting biological properties and are valuable compounds for supramolecular or medicinal chemistry [9]. This article provides a review study over the various natural acetophenone compounds that have been extracted and analyzed plants and microbial populations until January 2024.

## Search strategy

**Fig. 1:**
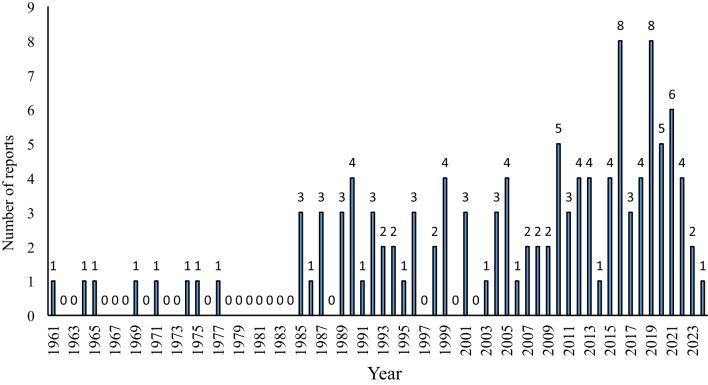
The number of published papers reporting the isolation of acetophenone derivatives since 1961

**Fig. 2:**
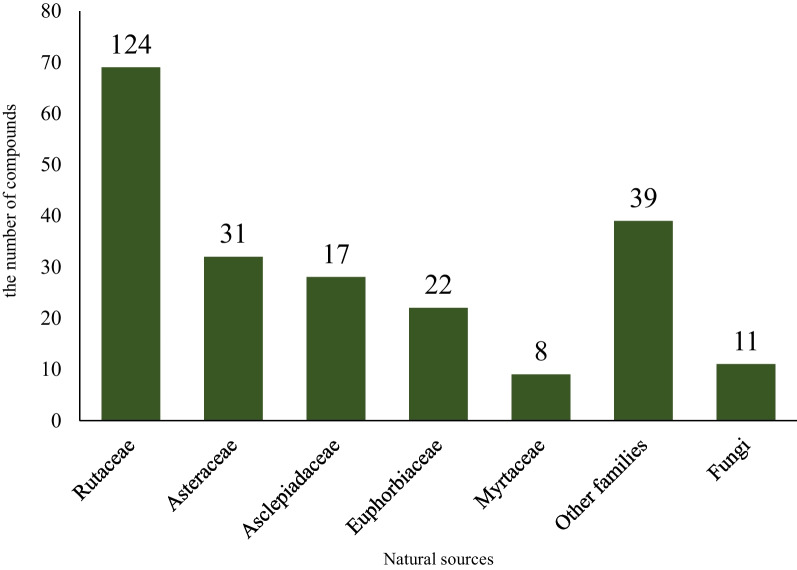
The number of acetophenone compounds isolated from natural sources including plants and fungi

An extensive survey of the “acetophenone”, “acetophenone derivatives”, and “biological activities” was conducted in scientific databases, including Scopus, Web of Science, PubMed, Google Scholar and Reaxys. The articles which included reports of novel isolated acetophenone were taken into account; however, the synthetic ones were excluded. Also, the reference lists of the included studies were manually investigated. Figure 1 illustrates the number of papers that have reported the isolation of novel acetophenone derivatives from natural resources. The number of isolated novel acetophenone compounds from different families of plants and fungi is also depicted in Fig. 2.

## Acetophenone derivatives produced by plants

### Rutaceae

Containing well over 2040 species categorized within around 170 genera, Rutaceae family, many members of which are aromatic plants [10] is well-known for its rich chemical profile that makes it the most chemically versatile plant family [11]. Species of Rutaceae have been used in the industries of gastronomy and perfumery and also in traditional medicine [10]. Regarding biological activities, the species of this family have displayed to possess antimicrobial, anticholinesterase, antidiarrheal, antileishmanial, larvicidal, antiprotozoal, fungicidal, and antioxidant activities [10].

#### The genus *Melicope*

**Fig. 3:**
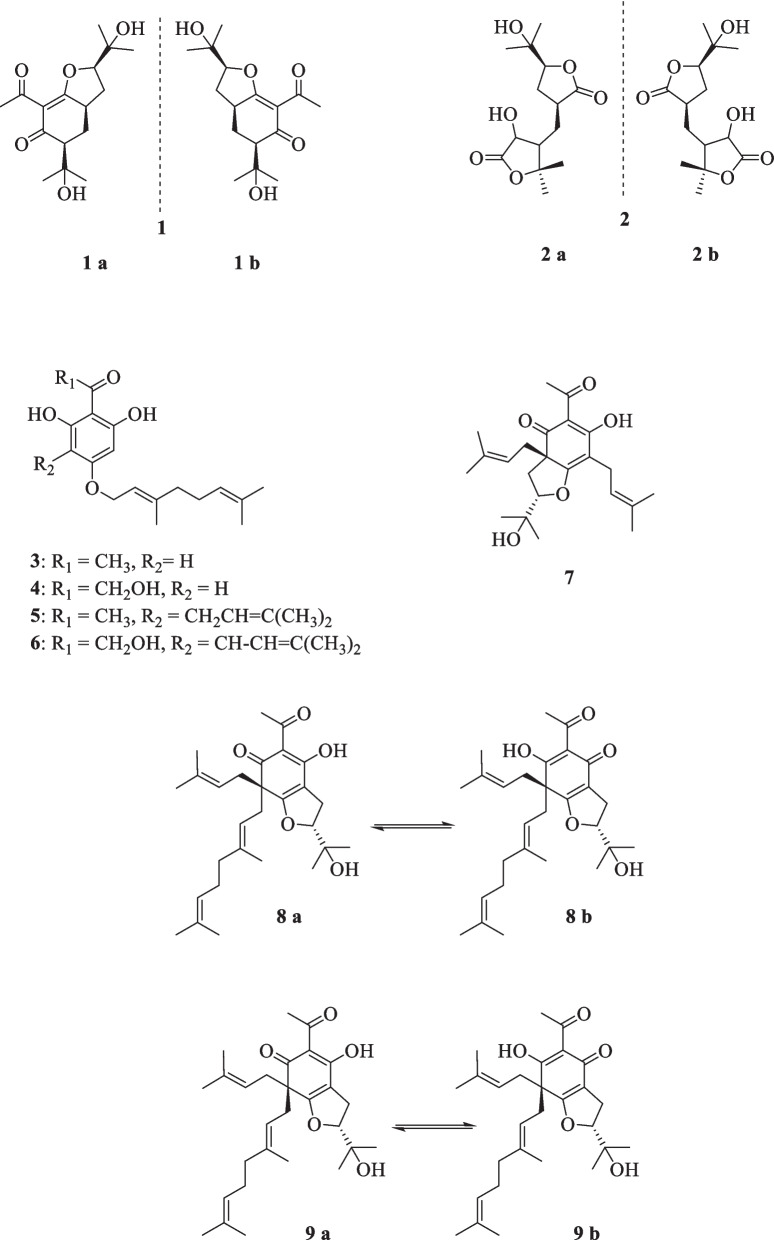

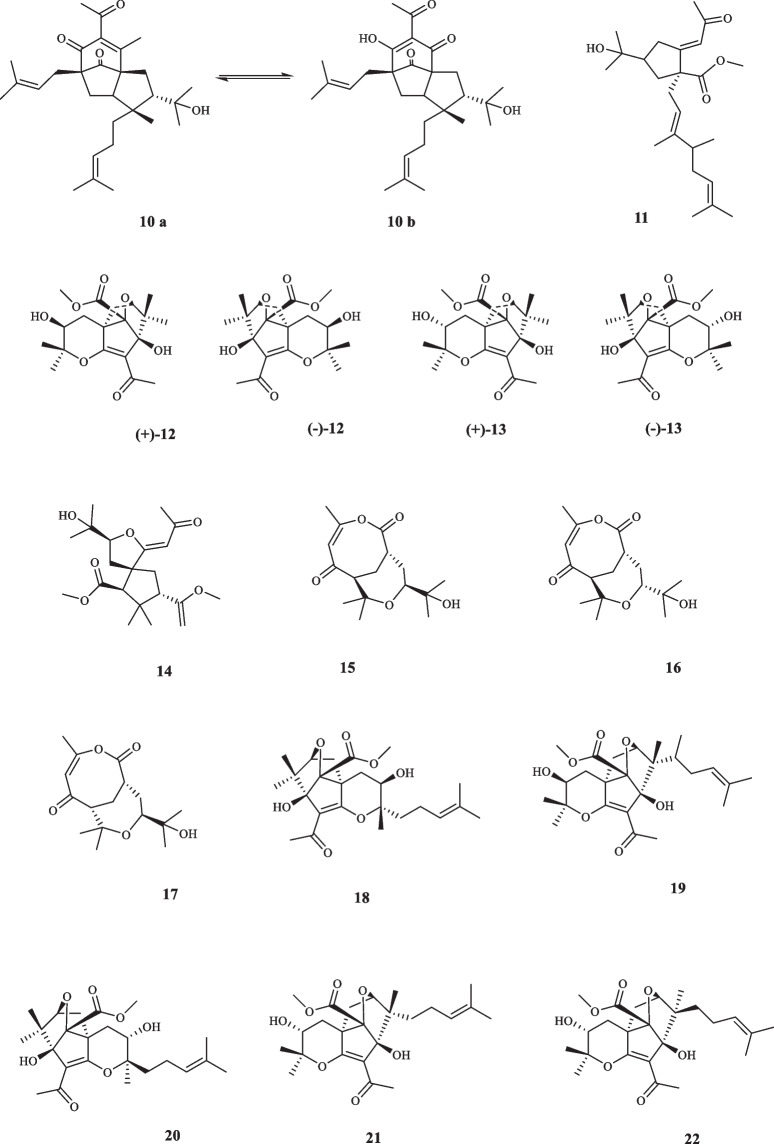

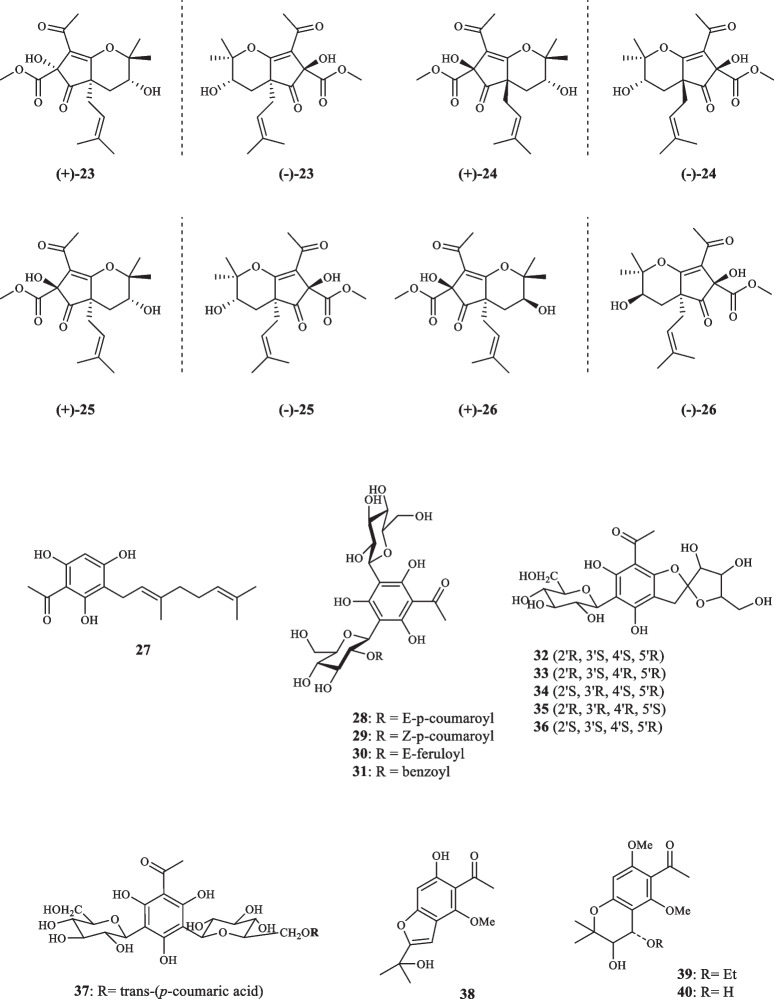

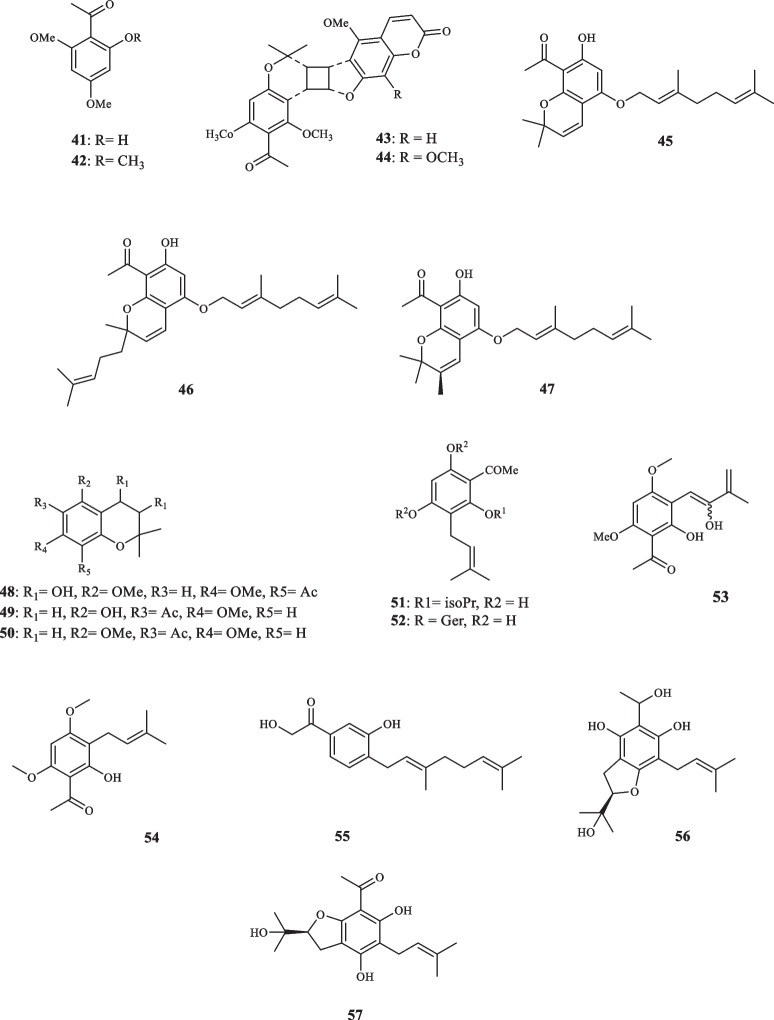

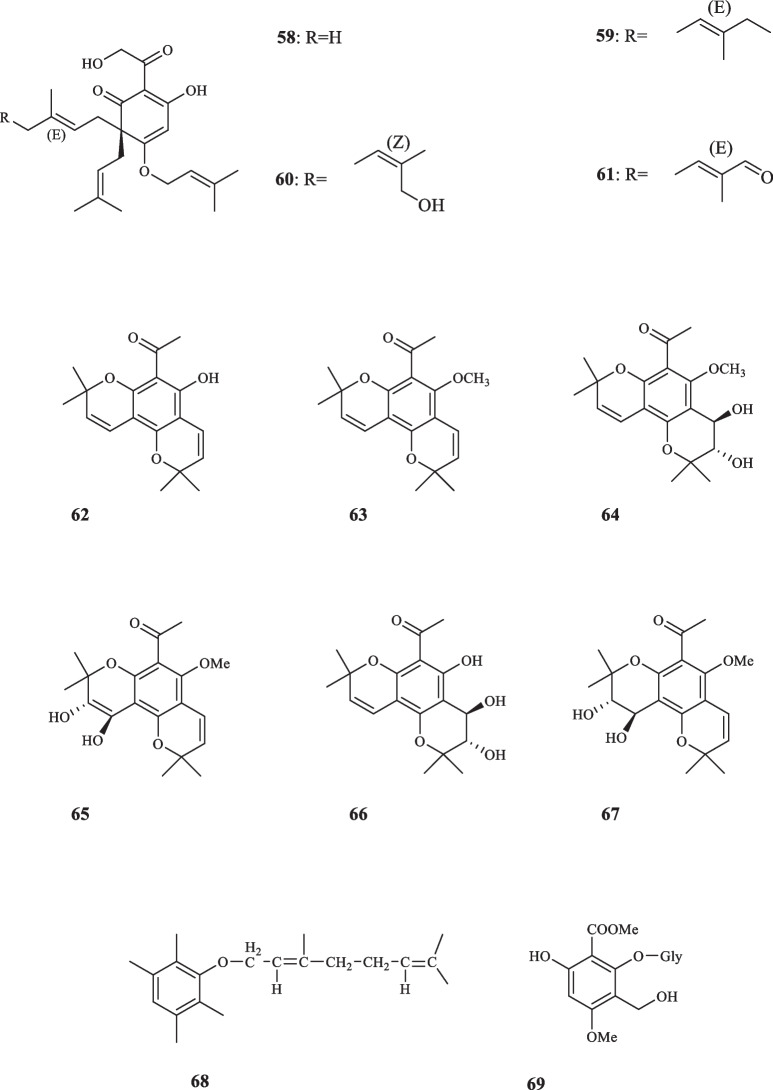
Acetophenone derivatives reported from *Melicope* species

Consisting of around 250 species, *Melicope* plants are scattered through the tropical regions of southern hemisphere [12]. Like genus *Acronychia*, the species of genus *Melicope* have been utilized for their therapeutic and healing properties during centuries [13]. The chemical diversity of *Melicope* species is owed to the presence of compounds such as flavonoids, benzopyrans, alkaloids, and acetophenones [14]. Prenylated acetophenone compounds, however, are the key compounds that constitute the chemotaxonomic traits of the genus [15]. Li et al. reported the isolation of meliviticine A (**1**) and meliviticine B **(2)** from *M. viticina* (16). The former (**1)** was identified to be a non-aromatic prenylated isopropylated acetophenone derivative; furthermore, the zero value of the specific optical rotation of **1** hinted to it being a racemic mixture. **2** was also figured to be an isopropylated rearranged prenylated acetophenone [16]. The application of subsequent chiral HPLC resolution led to the isolation of the two pairs of enantiomers (**1a** and **1b)** and **(2a** and **2b)** for** 1** and **2,** respectively [16]. **1** and** 2** were moderately effective against six strains of bacteria and fungi [16]. Adsersen et al. isolated and characterized two novel prenylated acetophenones, namely 2,6-dihydroxy-4-geranyloxyacetophenone (**3)** and 4-geranyloxy-2,6,b-trihydroxyacetophenone (**4**) from *M. obscura* and two acetophenones from *M. obtusifolia var. arborea*, namely 2,6-dihydroxy-4-geranyloxy-3-prenylacetophenone (**5**) and 4-geranyloxy-3-prenyl-2,6,b-trihydroxyacetophenone (**6**) [15]. Xu et al. extracted five acetophenone derivatives, three of which were with inseparable interconverting mixtures of tautomers from *M. pteleifolia*, specified as melicoptelin A (**7**), melicoptelin B1 (**8a**) and B2 (**8b**), melicoptelin C1 (**9a**) and C2 (**9b**), melicoptelin D1 (**10a**) and D2 (**10b**), and melicoptelin E (**11**) [17]. Prenylated acetophenone epimers, melicolone A (**12**) and melicolone B (**13**) were isolated from *M. pteleifolia*, followed by performing further chiral HPLC which yielded the entiomers ( +)- and (-)- of both compounds [18]. Nine more acetophenone derivatives, namely melicolones C-K (**14–22**) were also isolated from *M. pteleifolia* and examined for their drug resistance reduction characteristics [19]. **14–17** were identified to be as racemic mixtures and **18–22** were pure optically upon extraction. **18–22** boosted the cytotoxicity of doxorubicin with a reversal fold variating between 6.2 and 13.3 in a mixture with doxorubicin at the concentration of 5 µg/mL [19]. Xu et al. furthered isolated four new stereoisomer acetophenone compounds, evodialones A − D (**23–26**) from *M. pteleifolia* whose chiral-phase HPLC resolution resulted in the retrieval of eight enantiomers [20]. Shaari et al. investigated the core components of the extract of *M. pteleifolia champ* ex benth and identified 2,4,6-trihydroxy-3-geranylacetophenone (tHGA) (**27**) as the compound responsible for its attributed anti-inflammatory characteristics [21]. Upon the application on PBML 5-LOX human enzyme, **27** exerted an inhibitory activity with the IC_50_ value of 0.42 *µ*M. It also dose-dependently inhibited the LTC_4_ production with the IC_50_ value of 1.8 *µ*M with no inflicted cell toxicity [21]. Nakashima et al. succeeded in isolating four acetophenone derivatives assigned to as di-C-glycosides, pteleifolols A–D (**28–31**) from *M. pteleifolia* [22]. Studying on the same plant (*M. pteleifolia*), Nguyen et al. also isolated six more acetophenone derivatives, including five compounds with spiroketal-hexofuranoside rings, denoted melicospiroketal A-E (**32–36**) and one di-C-glycosidic phloroacetophenone, elucidated as 5-C-*β*-D-glucopyranosyl-3-C-(6-*O*-*trans*-*p*-*coumaroyl*)-*β*-Dglucopyranoside phloroacetophenone (**37**). The analysis of the extracted compounds indicated little to zero inhibitory effect against H1N1 influenza virus below the concentration of 400 *µ*M [23]. Parsons et al. introduced three new acetophenone derivatives extracted from the stem bark of *M. stipitata*, named furostipitol (**38**), 3β- hydroxydihydropyranostipitol-4⍺-ethyl ether (**39**), and 3,4-dihydroxydihydropyranostipitol (**85–40**) [24]. The screening of the extract of *M. borbonica* resulted in the isolation of xanthoxylin (**41**) and methylxanthoxylin (**42**), neither **41**, nor **42** possessed anti-inflammatory features against HeLa cells; regarding antifungal activities, however, both compounds proved effective. **41** and **42**, inhibited *Candida albicans* and *Penicillium expansum* with the MIA (minimum inhibitory amount) of 25 and 15 *µ*g, and > 50 and 20* µ*g, sequentially [25]. Xanthoxylin (**41**) has been proven to possess various pharmacological traits and potential applications. It has demonstrated anticancer properties, inhibiting the proliferation of oral squamous carcinoma cells, inducing apoptosis, autophagy, and cell cycle arrest [26]. Furthermore, xanthoxylin has shown promise as an agent targeting doxorubicin-resistant breast cancer cells, reducing their stemness and sensitizing them to doxorubicin [27]. Heterodimer compounds bearing acetophenone derivatives, namely meliquercifolin A (**43**) and meliquercifolin B (**44**) were found in the leaves of *M. quercifolia* [28]. **43** revealed to have strong cytotoxic activity against HeLa cancer cells with the IC_50_ value of 2.6 *µ*M, while **44** maintained ineffective in this regard. Neither **43** nor **44** exhibited any inhibitory effects against P-388 and MCF-7 cancer cells [28]. Chen et al. reported the extraction of three acetophenone compounds from the fruits of *M. semecarpifolia* (**45–47**) [14]. The anti-inflammatory qualities of isolated compounds were evaluated by assessing both the suppressing of fMLP/CB-induced superoxide anion and the release of elastase by human neutrophils. With respect to the first test, **45–47** exerted the IC_50_ values of 21.37, 23.24, and 30.61 µg/mL, respectively. Regarding the latter evaluation, the IC_50_ values of 27.35, 26.62, and 28.73 µg/mL were documented in the same order [14]. *M. lunu-ankenda* afforded three prenylated acetophenone derivatives, listed as 8-Acetyl-3,4-dihydroxy-5,7-dimethoxy-2,2-dimethylchroman (**48**), Isoevodionol (**49**), and Isoevodionol methyl ether (**50**) [29]. Phenylethanones acetophenones (**51–52**) were also extracted from *Euodia lunu-ankenda* [30]. Le et al. detected the presence of two newly-discovered acetophenone compounds, namely melibarbinon A (**53**), and melibarbinon B (**54**) from *M. barbigera*. The cytotoxic availability of **54** was assessed against A2780 cell lines, which were suppressed at the IC_50_ value of 30 *µ*M [31]. Vu et al. isolated three acetophenone derivatives, namely patulinones E − G (**55–57**) from *M. patulinervia*. The experiment regarding the inhibitory activity of the isolated compounds against α-glucosidase indicated the IC_50_ values of 41.68, 6.02, and 67.44 *μ*M, attributed to **55–57,** respectively [32]. Simonsen isolated four non-aromatic acetophenone compounds, named coodeanone A (**58**), coodeanones E-B (**59**), coodeanones Z-B (**60**) and coodeanone C (**61**) from *M. coodeana* [12]. Coodeanone B could be detected in the configuration of either E or Z; hence, being counted as two acetophenone derivatives [12]. The extract of *M. erromangensis* revealed to contain six novel acetophenone derivatives (**62–67**) [33]. Acetophenone compounds, refered to by the trivial names of melicopol (**68**) and methylmelicopo (**69**) were isolated from the bark of *M. broadbentiana* [34]. Isolated Acetophenones (**1–69**) from the genus *Melicope* are depicted in Fig. 3.

#### The genus *Acronychia*

The genus *Acronychia* is comprised of 44 species, distributed mainly along Asia and Australia [35]. The various parts of these plants including roots, leaves, stems, and the fruits have a wide range of medical applications such as mitigating diarrhea, asthma, itchy skin, cough, scales, hemorrhage, fever, etc. [36]. *Acronychia* species are also utilized for the treatment of fungal infection, spasm, pyrexia, stomachache, and rheumatism [35]. The species of the genus *Acronychia* have been a rich source of bioactive compounds including flavonoids, quinoline, lignans, steroids, coumarins, triterpenes, acridone alkaloids, and acetophenones [35]. Apart from owning therapeutic characteristics, different part of *Acronychia* plants have had other usages as their essential oil (EO) is used in cosmetics and their aerial parts are used as food and condiments [35].

The chemical compounds of *Acronychia oligophlebia* were studied by Chen et al. and seven new acetophenone-derived compounds were identified. These compounds, named acrolione A-G (**70–76**) were all discerned to be responsible for the antioxidant activities of their host plant as they were elucidated to possess the pertaining effects, using DPPH radical-scavenging capacity and FRAP assays. As regards the anti-inflammatory characteristics, **70**, **72**, **73,** and **74** proved to be effective at the IC_50_ values of 26.4, 46.0, 79.4, and 57.3 *µ*M against RAW 264.7 cells, respectively [37]. Three prenylated acetophenone derivatives, called acronyculatin (P-R) (**77–79**), were also extracted from *A. oligophlebia* by Niu et al. [16]. The cytotoxic activity of **77–79** against MCF-7 cancer cells was tested, which resulted in the inhibitory effect at the IC_50_ values of 56.8, 40.4, and 69.1 *µ*M, respectively [16]. Yang et al. did conducted another investigation on *A. oligophlebia* which resulted in the isolation of six new acetophenone derivatives from the leaves of the plant (**80–85**). The cytotoxic activity of **81–85** were evaluated against MCF-7 cancer cells. **81** and **85** exhibited moderate inhibitory activities with the IC_50_ values of 33.5 and 25.6 *µ*M, respectively; whereas **82–84** exerted weak effects with the IC_50_ values of 80.2, 71.1 and 46.3 *μ*M, in the same order [38].

**Fig. 4:**
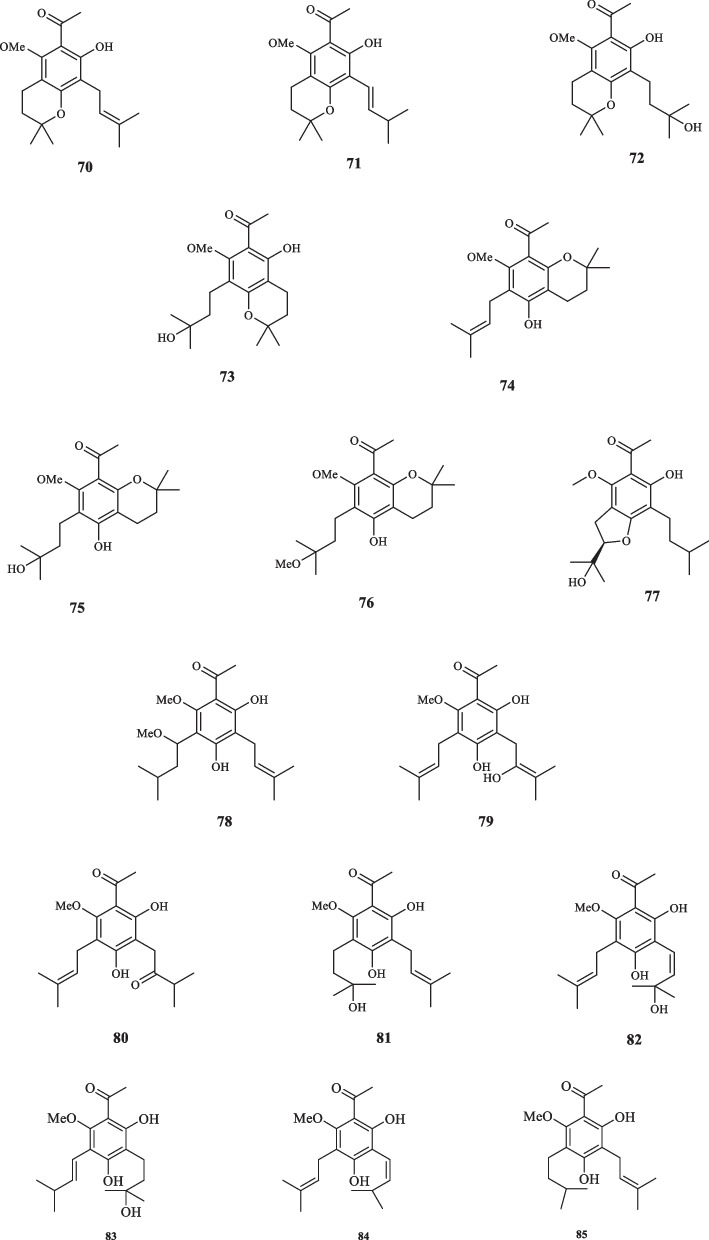

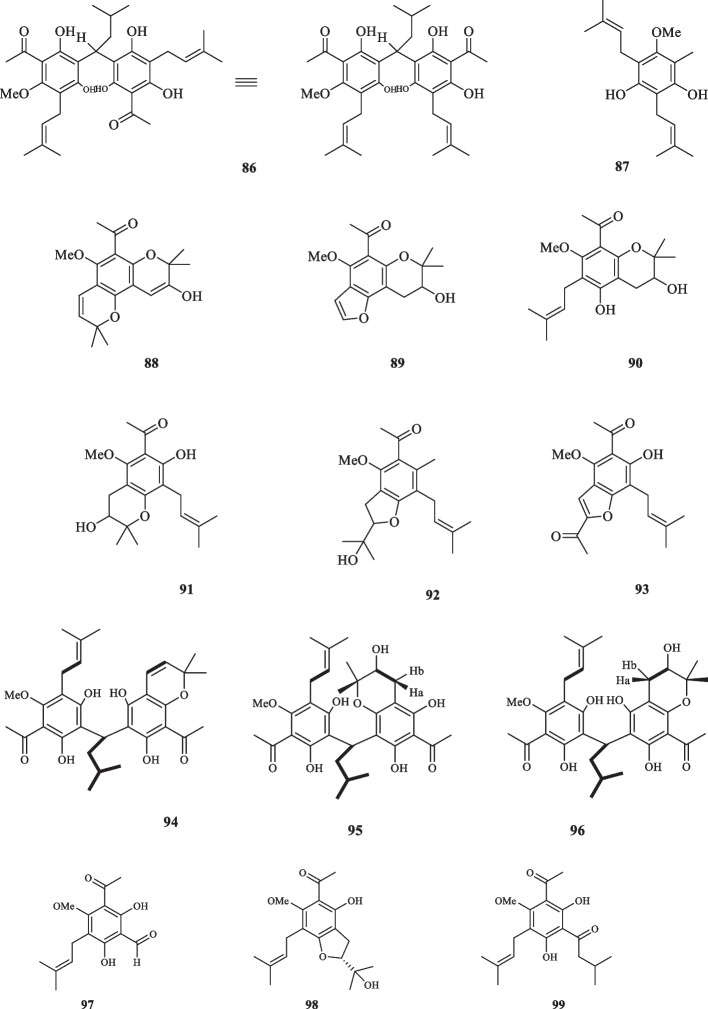

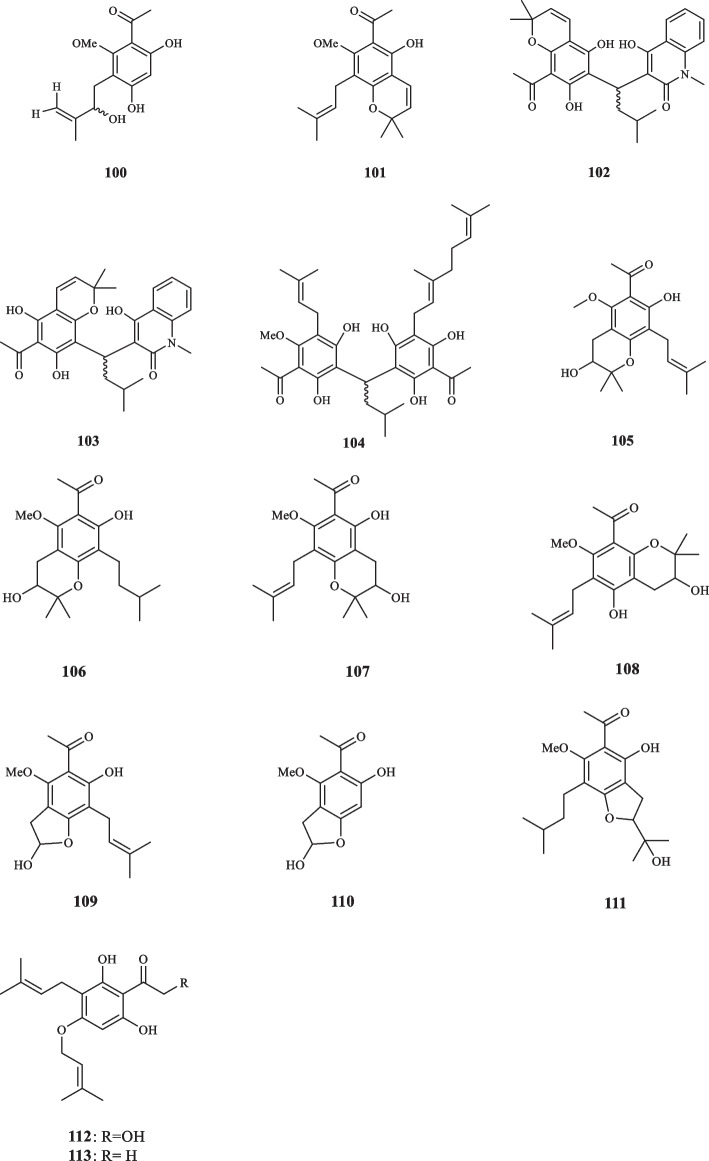
Acetophenone derivatives reported from the genus *Acronychia*

Acrovestone (**86**) was extracted and structurally elucidated from *A. pedunculata* by Wu et al. [39]. This compound displayed potent cytotoxic activity by exerting total inhibition at the concentration of 0.5 µg/mL in human KB tissue culture. It also demonstrated strong cytotoxicity against A-549, L-1210, and P-388 cancer cells at the ED_50_ values of 0.98, 2.95, and 3.28 µg/mL, respectively [39]. The continuation of examination on *A. pedunculata* led to the extraction of an undescribed arylketone acetophenone (**87**) [40]. Ito et al. discovered three novel acetophenone compounds, namely acrophenones A-C (**88–90**), in *A. pedunculata*, all of which failed to inhibit the growth of five leukemia cell lines (NALM6, Jurkat, HPB-ALL, K562, and PBMNC [41]. In pursuit of exploiting the chemical components of *A. pedunculata* for cancer prevention application, three more acetophenone derivatives were extracted from *A. pedunculata* by Ito et al., denoted acrophenones D-F (**91–93**) [42]. Kouloura et al. isolated three more acetophenone dimers from *A. pedunculata* and elucidated them as: acropyrone (**94**), acropyranol A (**95**), and acropyranol B (**96**). It is noteworthy that prenylated acetophenone dimers are found exclusively in the genus *Acronychia* [43]. The continuation of research on *A. pedunculata*, led to the isolation of five more acetophenone compounds by Su et al. acronyculatins A-E ( **97–101**) [44]. **77** was also discovered to be in the chemical profile of *A. pedunculata* [45]. Upon the application on murine leukemia P-388 cells, **77** displayed an inhibitory effect with the IC_50_ value of 15.42 *µ*M [45]. Acetophenone compounds, assigned as acroquinolones A-B (**102** and **103**), belonging to a class of acetophenone-alkaloid hybrids were extracted from *A. pedunculata (L.)* Miq. These compounds were tested against a group of cancer cell lines and proved to exhibit minor inhibitory effects against A549 and HCT116 and moderate cytotoxicity against HT29 and HeLa with the IC_50_ values of 21.8 and 14.2 µg/mL, respectively [46]. Nathabumroong et al. isolated an isoprenylated acetophenone, named 5’-prenylacrovestone (**104**) from *A. pedunculata* [47]. Seven acetophenone monomers, named acronyculatins I − O (**105–111**) were detected and extracted from *A. trifoliolata* by Miyake et al. The isolated compounds were evaluated for their antiproliferative properties against five lines of human cancer cells specified as A549, KB, KB-VIN, MDA-MB 231, and MCF-7. While **105** and **106** caused their corresponding inhibitory effects at the IC_50_ values of 26.6, 25.6, 19.2, > 40, and 30.8 *µ*M (**105**), and 19.9, 20.4, 16.2, 22.6, and 19.4 *µ*M (**106**), respectively, the remainder of isolated compounds exhibited IC_50_ values excessing 40 *µ*M for the collective cell lines [48]. *A. crassipetala* was elucidated to host two prenylated acetophenones, namely crassipetalonol A (**112**) and crassipetalone A (**113**). The latter **(113)** had been previously detected in *Euodia lunu-ankenda*, along with the report of its fungicide activity [30], and *Urtica dioica L..* **112** also showed to possess high levels of toxicity and little to none antibacterial traits when tested at the high concentration of 156 *µ*M against ESKAPE pathogenes [49]; Comparatively, **113** elucidated to have strong antibacterial activity as it inhibited *Entercoccus faecium* and Gram-positive bacteria, *S. aureus* at the MIC_75_ values of 2.6 and 20.6 *µ*M, respectively [49]. The derived acetophenone compounds from the genus *Acronychia* are illustrated in Fig. 4.

### Other Rutaceae species

**Fig. 5:**
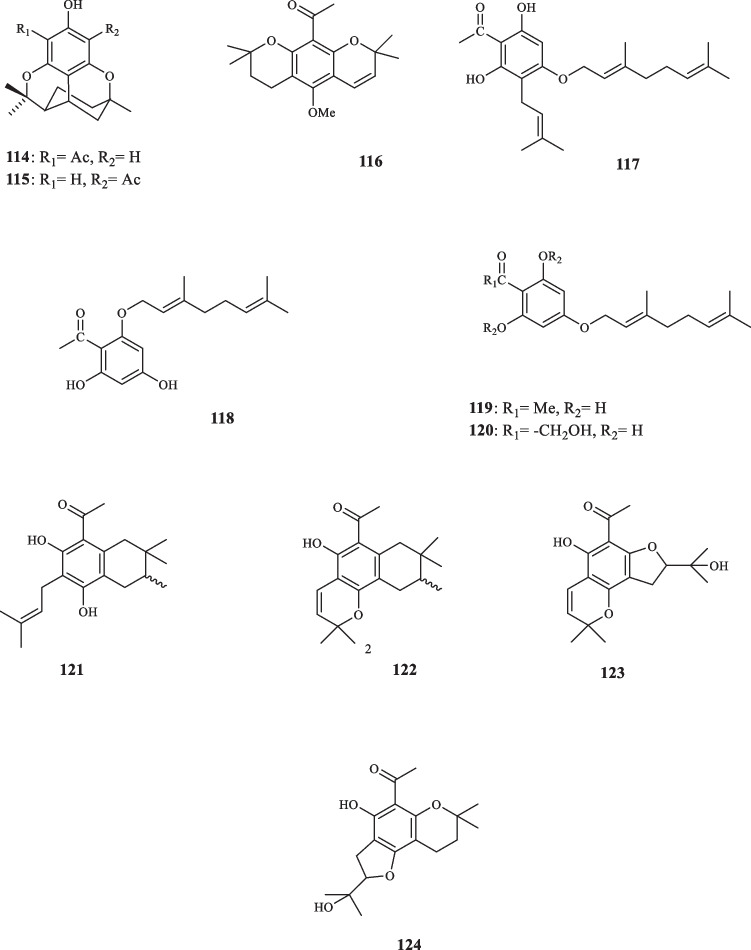
Acetophenone derivatives reported from other Rutaceae species

Several actephonones were isolated from other species of Rutaceae (Fig. 5). Goh et al. introduced two phloroacetophenone derivatives, namely melifolione 1a (**114**) and melifolione 1b (**115**) from *Euodia latifolia* [50]. The leaves of *Bosistoa euodifoumis* afforded the prenylated acetophenone derivative, called franklinone (**116**) [51]. Chou et al. extracted acetophenone derivatives, identified as 4-(1'-geranyloxy)-2,6-dihydroxy-3-isopentenylacetophenone (**117**), 2-(1'-geranyloxy)-4,6-dihydroxyacetophenone (**118**), 4-(1'-geranyloxy)-2,6-dihydroxyacetophenone (**119**), and 4-(1'-geranyloxy)-P,2,6-trihydroxyacetophenone (**120**) from *E. merrillii* [52]. Hartmann and Nienhaus discovered an acetophenone compound, xanthoxylin (**41**), that was extracted from the bark of *Citrus limon*, infected two strains of fungi, *Hendersonula toruloidea* and *Phytophthora citrophthora*. Having been absent in the healthy barks of *Citrus lemon*, the lesion-tissue-extracted **41** was found trice the concentration it had when inhibiting the growth of the pertaining fungi in vitro at the ED_50_ value of 0.8 mM. The concentration of **41**, also, peaked in the dead tissue [53]. Four novel prenylated acetophenone compounds were isolated from *Bosistoa selwynii* identified as selwynone (**121**), pyranoselwynone (**122**), furanoselwynone (**123**), and isofuranoselwynone (**124**) [54]. Quader et alextracted and characterized acetophenones **41** from *Acradenia frankliniae* [55].

### Asteraceae

**Fig. 6:**
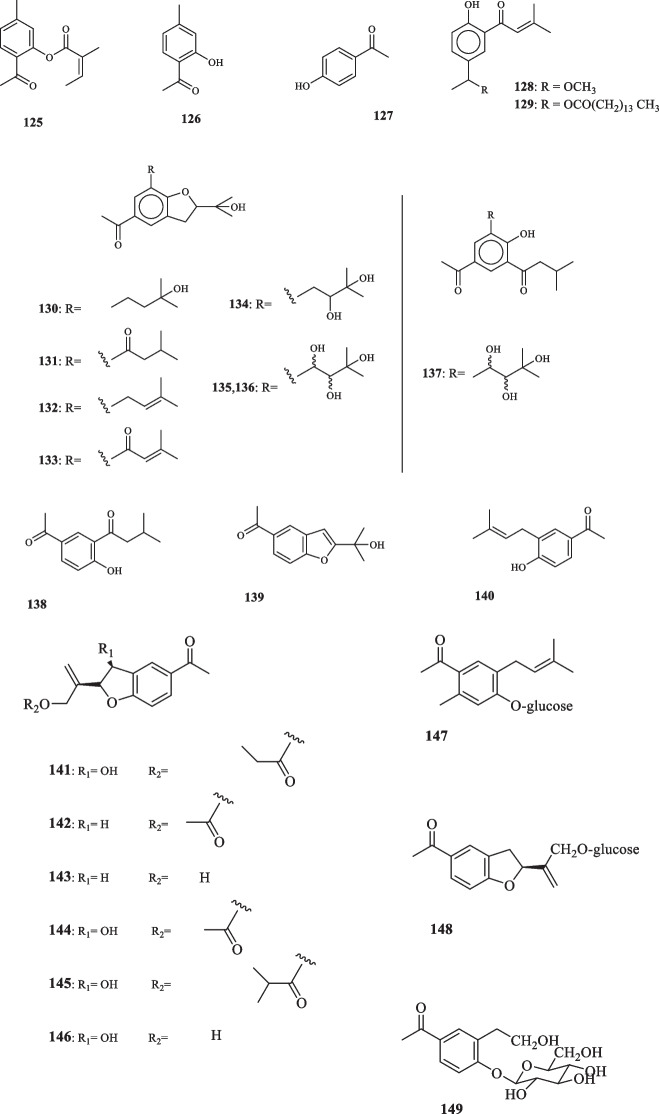

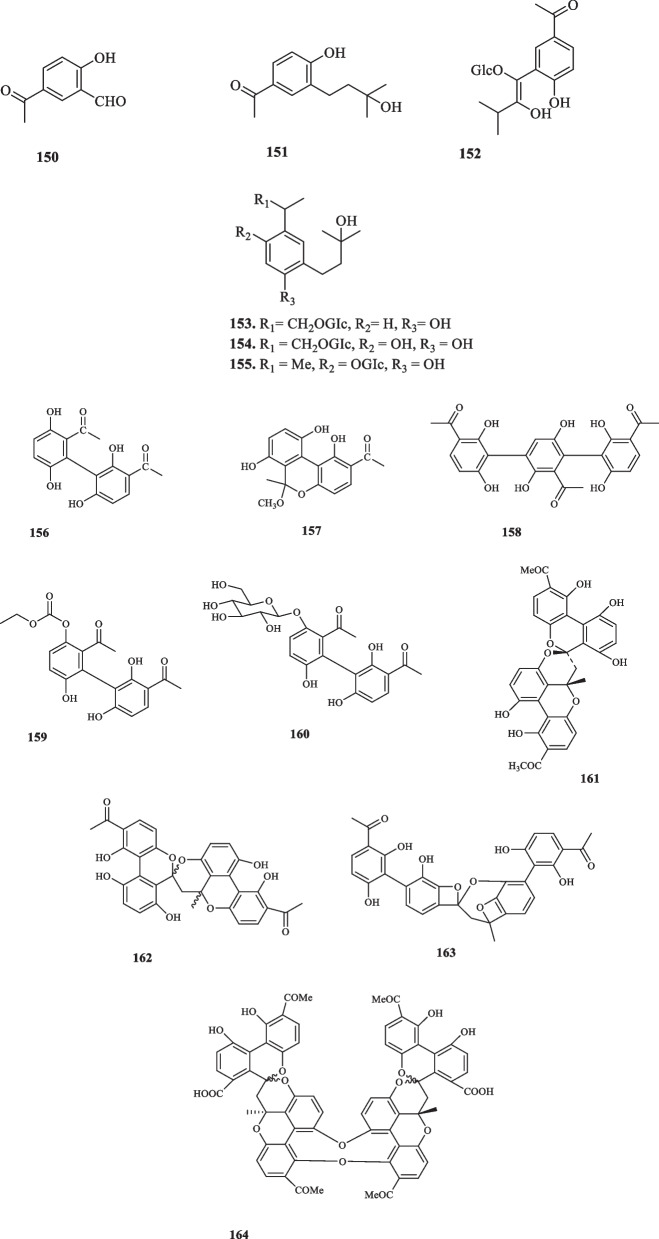
Acetophenone derivatives reported from the family Asteraceae

Titled the biggest family of flowering plants, Asteraceae is comprised of more than 1600 genera and 25,000 species scattered around the world [56]. Most species, however, are present more densely in the arid and semi-arid regions of subtropical areas [57]. Members of Asteraceae have been long used for their medicinal traits such as antipyretic, hepatoprotective, smooth muscle relaxant, laxatives and their ability to heal flatulence, lumbago, hemorrhoids, etc. Furthermore, the anti-oxidant and anti-inflammatory activities of the members of this family are well-acknowledged [57]. Therefore it can be deduced that the majority of Asteraceae members are categorized as medicinal plants, owing to their rich chemical profile, including flavonoids, mucilage, tannins, glycosides, and carbohydrate [57]. The presence of more phytochemical components, namely lignans, polyphenolic compounds, sterols, phenolic acids, diterpenoids, polyphenols and saponins has also been reported to contribute to their therapeutic effects [58]. Acetophenones discovered in some genera of Asteraceae have proven to contribute to the pertaining properties of the family species. Embarking on the attempt to explore the cytotoxic constituents of *Eupatorium fortune*, Chang et al. isolated an acetophenone derivative, known as eupatofortunone (**125**) that was elucidated to contribute to the therapeutic values of the host plant [59]. 2-Hydroxy-4-methylacetophenone **(126)** [60] was also extracted during the process. Compounds **125** and **126** were assayed as to their ability to suppress the proliferation of MCF-7 and A549 cells. Regarding **125**, the inhibition was observed at the IC_50_ value of 82.15 and 86.63 *µ*M, respectively. In another study, **126** showed no the antiproliferation effect (IC_50_ values of > 100 *µ*M) for both cell lines [59]. Trang et al. also extracted **126** from the aerial parts of *E. stoechadosmum* [60]. Mendes do Nascimento et al. obtained two *p*-hydroxyacetophenone (**127**) derivatives from *Calea uniflora* and denoted them 2-senecioyl-4-(methoxyethyl)-phenol (**128**), and 2-senecioyl-4(pentadecanoyloxyethyl)-phenol (**129**). p-Hydroxyacetophenone (**127**) has shown various pharmacological activities and potential applications. It has been found to possess hepatoprotective, antioxidative, and anti-inflammatory properties, making it a potential treatment for alcoholic liver disease. It has also demonstrated antioxidative, antinociceptive, and anti-inflammatory effects, suggesting its therapeutic potential in inflammation-associated diseases [61]. Both **128** and **129** exhibited trypanocidal activities against *Trypanosoma cruzi* parasite. At the administration doses of 100, 250, and 500 µg/mL, **128** and **129** inflicted lysis on *Trypanosoma cruzi* at the percentage-wise records at three different concentrations of 27.5 (10 µg/mL), 28.9 (250 µg/mL), 42.0 (500 µg/mL), and 8.8 (10 µg/mL), 24.7 (250 µg/mL), and 70.9 (500 µg/mL), respectively [62]; furthermore, **128** and **129** both displayed antifungal traits against four strains of *Candida* spp, namely *C. albicans*, *C. krusei*, *C. parapsilosis*, *C. glabrata*, and four dermatophytes including two strains of *Trichophyton rubrum* (Tr-5 and Tr-19), and two strains of *Trichophyton mentagrophyte* (Tm-9 and Tm-17). Both **128** and **129** exerted fungitoxicity against all the strains of dermatophytes with the MIC value of 1000 µg/mL. While both compounds were ineffective against inhibiting *C. krusei* and *C. parapsilosis*, both **128** and **129** suppressed *C. albicans* at the same MIC value of 500 µg/mL, and **128** deterred the proliferation of *C. glabrata* with the MIC value of 500 µg/mL [62]. The aerial parts of *Ophryosporus macrodon* yielded eight novel diprenylated *p*-hydroxyacetophenone derivatives (**130–137**). 136 and 137 were elucidated to be threo and erythro isomers, respectively [63]. 4'-Hydroxy-3'-(3-methylbutanoyl)acetophenone (**138**) was isolated from *Flourensia cernua* by Bohlmann and Grenz [64] and from *Polymnia sonchifolia* by Takasugi and Masuda [65]. 5-Acetyl-2-(1-hydroxy-lmethylethyl)benzofuran (**139**) was also isolated in this assay. Thomas-Barberan et al. identified 4-hydroxy3(isopentent-2-yl) acetophenone (**140**) from *Helichrvsum italicum*, and revealed its antibacterial activity against gram positive bacteria (*Bacillus sp*. and *Staphylococcus epidermidis*) and *E. coli* gram negative bacteria at the MIC values of > 100 and 25 ug/mL, respectively [66]. **140**, also, exhibited antifungal quality by inhibiting 5 strains of fungi (*Cladosporium herbarum*, *Phyrhophthora capsica*, *Neurospora crassa*, *Penicillium italicurn*, and *P. digitalum*) at the MIC values of 10, 50, 100, 100, and 50 µg/mL, respectively [66]. Takasugi and Masuda also extracted **140** from *Polymnia sonchifolia* [65]. The examination of *H. italicum* with respect to its constituting compounds led to the identification of a new acetophenone derivative, named gnaphaliol 9-*O*-propanoate (**141**) [67]. Rigano et al. [67] isolated acetoxytremetone (**142**), 10-hydroxytremetone (**143**), and 1-[2-[1-[(acetyloxy)methyl]ethenyl]-2,3-dihydro-3-hydroxy-5-benzofuranyl]-ethanone (**144**) from this plant. Rigano et al. [67], also identified 13-(2methylpropanoyloxy)toxol (**145**), and gnaphaliol (**146**) in *H. italicum* that had been previously isolated from *Diplostephium cinereum* [68], in addition to being extracted from *Gnaphalium polycaulon* [67, 69]. Compounds **141**, **142**, and **144** were tested for evaluation of their biological effects, namely anti-inflammatory and antioxidant properties. Regarding the former, none of the aforementioned compounds proved to have such an effect; and only **142**, which was previously denoted for its spasmolytic activity [70]**,** showed to possess antioxidant activities [67]. Sala et al., also, isolated three previously-unidentified acetophenone glucosides, (**147**–**149**) [71] from *H. italicum* and reported the anti-inflammatory effects of them on TPA-induced mouse ear edema, where **147, 148,** and **149** managed to reduce the ear thickness by values approximating roughly 200 *µ*m [71]. *Senecio graveolens* is another member of Asteraceae whose biochemical profile was shown to host two novel **127** derivatives, namely 5-acetylsalicylaldehyde (**150**) and 4-hydroxy-3-(3′’-hydroxyisopentyl)acetophenone (**151**) [72]. The last novel acetophenone compounds from the family Asteraceae were extracted from *Helianthus annuus L*., elucidated as 4-hydroxy-3-(2′-hydroxy-3′-methyl-1′-butenyl) acetophenone-1′-O-β-dglucopyranoside (**152**), 4-hydroxy-3-((Z)-3′-hydroxy-3′-methyl-1′-butenyl) acetophenone-8-O-β-D-glucopyranoside (**153**), 4,6-hydroxy-3-((Z)-3′-hydroxy-3′-methyl-1′-butenyl) acetophenone-8-O-β-D-glucopyranoside (**154**), and 4-hydroxy-3-((Z)-3′-hydroxy-3′-methyl-1′-butenyl) acetophenone-6-O-β-D-glucopyranoside (**155**) [73]. None of these compounds showed to possess any significant cytotoxicity upon their application on MH-S cells. Furthermore, these compounds displayed no noticeable anti-inflammatory activities with regard to the inhibition of NO secretion at either 12.5 or 25 *µ*M concentrations. Acetophenone compounds, extracted from the family Asteraceae are elucidated in Fig. 6.

### Asclepiadaceae

**Fig. 7:**
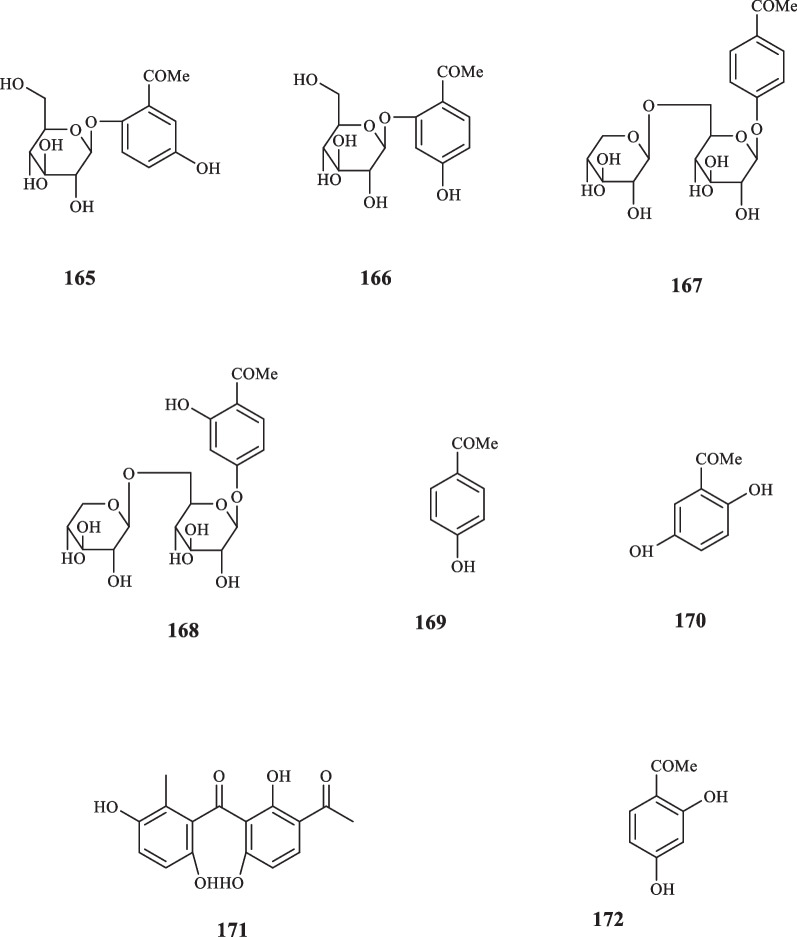
Acetophenone derivatives reported from the family Asclepiadaceae

The biggest genus of the family is *Cynanchum* L., the species of which have been used in traditional medicine for the treatment of various diseases and disorders [74]. The phytochemical components of those species account for their immune regulation, anti-tumor, and anti-oxidation properties [75]. Hwang et al. extracted two acetophenone derivatives, cynandione A (**156**) and cynanchone A (**157**), from the roots of *Cynanchum wilfordii*, neither of which showed any multidrug-resistance [76]. In another study on the components of the root bark of *C. wilfordii*, three acetophenone derivatives, namely cynwilforones A-C (**158–160)** were discovered by Jiang et al. [77]. **158** reportedly exhibited hypoglycemic effects in the primary hepatocytes of mice by inhibiting hepatic gluconeogenesis through down-regulating the expression of G6P and PEPCK enzymes, which are responsible for the control of gluconeogenesis [77]. The application of two doses of **158** at 20 *µ*M and 40 *µ*M yielded the suppression of hepatic gluconeogenesis by 12.5% and 29.4%, respectively; hence, positing the potential healing traits of the root barks of *C. wilfordii*, along with their current use for mitigating neurasthenia, abscesses, impotency, and lumbago [77]. Cynandione A (**156**) and its dimers, cynandiones B & C (**161** and **162**) were isolated from *C. taiwanianum* [78]. In continuation of this study, one more acetophenone derivative was identified and extracted from *C. taiwanianum*, named cynanchone D (**163**) [78]. Cynanchone A (**157**) was also isolated in this assay [78]. A cytotoxic acetophenone, called cynantetrone (**164**) was isolated from *C. taiwanianum* by Huang et al. [79]. The assessment for its bioactivity revealed a cytotoxic trait against PLC/PRF/5, and T-24 cancer cell lines with the ED_50_ values of 6.6 and 3.5 µg/mL. The inhibitory effect of **164** on KB cell lines was proven to be insignificant [79]. Having investigated *C. bungei*, Li et al. isolated four acetophenone glucoside compounds which were regarded to as bungeisides A-D (**165–168**) [80]. This species later exhibited to possess four more novel acetophenone compounds identified as 4-hydroxyacetophenone (**169**), 2,5-dihydroxyacetophenone (**170**), baishouwubenzophenone (**171**), and 2,4-dihydroxyacetophenone (**172**) [81]. **169** was also later discovered in *C. auriculatum* and *C. wilfordii* [82–84]. Figure 7 shows the structures of extracted acetophenones from Asclepiadaceae.

### Euphorbiaceae

**Fig. 8:**
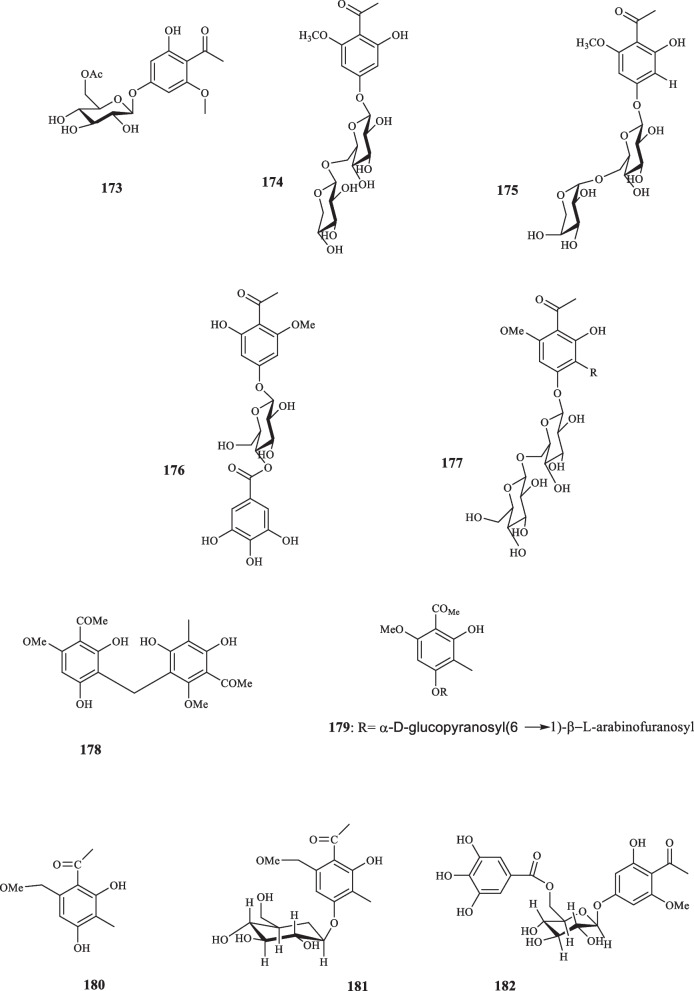

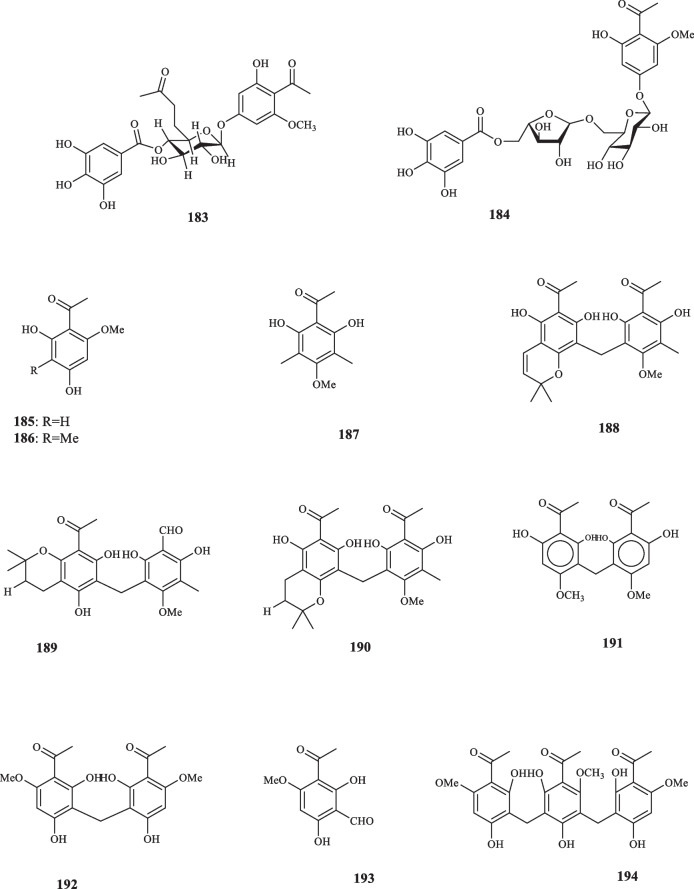
Acetophenone derivatives reported from the family Euphorbiaceae

Euphorbiaceae contains 299 genera and 8000 species, grouped into seven subfamilies, listed as Euphorbioideae, Crotonoideae, Acalyphoideae, Cheilosoideae, Peroideae, Oldfieldioideae, and Phyllanthoideae, which are found in the forms of trees, shrubs, and annual plants [85]. The species belonging to Euphorbiacea family are best grown in tropical and subtropical regions and rarely grow in cold temperate climates [86]. *Euphorbia* is the vastest genus, containing over 200 species. The members of this family are defined among the most phytochemically diverse species [87]. Alkaloids, tannins, diterpenes, cyanogenic glycosides, triterpenes, and glucosinolated lipids are some of the significant secondary metabolites, reported from the species of Euphorbiaceae [86]. Du et al. extracted the previously-unknown acetophenone glycoside, 2-hydroxy-6-methoxyacetophenone-4-*O*-(6′-acetate)-β-D-glucopyranoside (**173**) from *E. fischeriana* [88]. **173** elucidated to possess antiproliferative activity against AGS (IC_50_ = 39.85 *µ*M) and Hep-G2 (IC_50_ = 35.06 *µ*M) cancer cell lines [88]. Three acetophenone glycosides, (**174–176**) were also isolated from *E. fischeriana* by Huang et al. [89]. The iosolated compounds were ineffective against a group of human cancer cell lines (MCF-7, LoVo, SH-SY5Y, U87, U118, and U251) with the IC_50_ values of more than 100 *µ*M [89]. Wang et al. obtained an acetophenone glycoside denoted (**177**) from *E. ebracteolate* (91). By exploiting the DPPH scavenging assay, **177** was discovered to possess antioxidant activity at the IC_50_ value of 34.62 µg/mL [90]. Yin et al. isolated two acetophenone derivatives (**178** and **179)** from *E. ebracteolate* [91]. Acetophenone compounds, **(180** and **181)** were extracted from *E. ebracteolata Hayata* [92]. The cytotoxicity activity of **180** and **181** were assessed against four lines of human cancer cells, namely Hela-60, MCF-7, A-549, and SMMC-7541, which elucidated the following results: **180** hindered the cell lines with the IC_50_ values of 0.095, 6.85, 8.71, and 16.52 μg/mL in the same order, whereas **181** left its cytotoxic effects at the IC_50_ values of 2.69, 0.346, 0.879, and 12.86 μg/mL, respectively [92]. Acetophenone glycosides: langduphenone A (**182)**, langduphenone B** (183)**, and langduphenone C** (184)** were extracted from *E. fischeriana* [93]. Compounds **182**–**184** were elucidated to possess inhibitory qualities against cancer cells and bacteria. Regarding the former, the isolated compounds were applied to five lines of cancer cells including Hep-G2, Hep-3B, A549, NCI–H460, and AGS; **182** indicated the cytotoxic effects with the IC_50_ values of 33.9, 30.2, 39.4, 27.3, and 44.8 *µ*M, respectively. **183** and **184**, too, displayed the same trait at the sequential values of 27.8, 25.4, 37.2, 31.6, 38.2* µ*M and 45.4, > 50, 37.2, 36.4, > 50 *µ*M. As for the antibacterial activity, two Gram-negative bacteria, *E. coli P. aeruginosa*, and two strains of Gram-positive bacteria, i.e., *S. aureus* and *B. subtilis* were used for the measurement; compounds **182**–**184** inhibited the proliferation of the four aforementioned bacteria with the ternary MIC values listed as: (12.5, 6.25, 6.25), (6.25, 12.5, 3.12), (6.25, 3.12, 1.56), and (6.25, 3.12, 3.12) µg/mL, respectively [93]. Sun and Liu elucidated the structure of 2,4-dihydroxy-6-methoxy-1-acetophenone (**185**) and 2,4-Dihydroxy-6-methoxy-3-methylacetophenone (**186**) [94] which were originally isolated from *E. fischeriana* by che et al. [95] and liu et al. [96]. Research into the phloroglucinol derivatives of *Mallotus japonicus* gave rise to the identification of a new acetophenone compound, called mallophenone (**187**), and three of its derivatives, namely isomallotochromene (**188**), mallotochroman (**189**), and isomallotochroman (**190**). The cytotoxicity of **187**–**190** against HeLa cancer cells were measured and their ID_50_ values were registered respectively as follows: 14.80, 0.28, 49.10, and 8.80 µg/mL. **198**–**201**, also, had the Anti HSV-1 activities with the in-turn ED_50_ values of 6.18, 0.11, 48.00, and 0.97 µg/mL, respectively [97]. From the fruits of *Mallotus japonicus*, Ariswa et al. identified another acetophenone derivative, called mallotophenone (**191**) [98]. Having been tested against Leukemia L-5178Y cells of mice in vitro, compound **191** performed inhibitory activity with the ED50 value of 6.10 µg/mL [98]. Regarding the toxicity in the KB system, **191**, also, showed potent with the ED50 value of 2.40 µg/mL [98]. The acetone extract of the roots of *E. kansui* provided two acetophenone derivatives (**192** and **193**) [99]. Geng et al. identified an acetophenone trimer from *E. ebracteolate* and assigned it as ebracteolatain C (**194**) [100]. Euphorbiaceae’s derived acetophenone derivatives are illustrated in Fig. 8.

### Myrtaceae

**Fig. 9:**
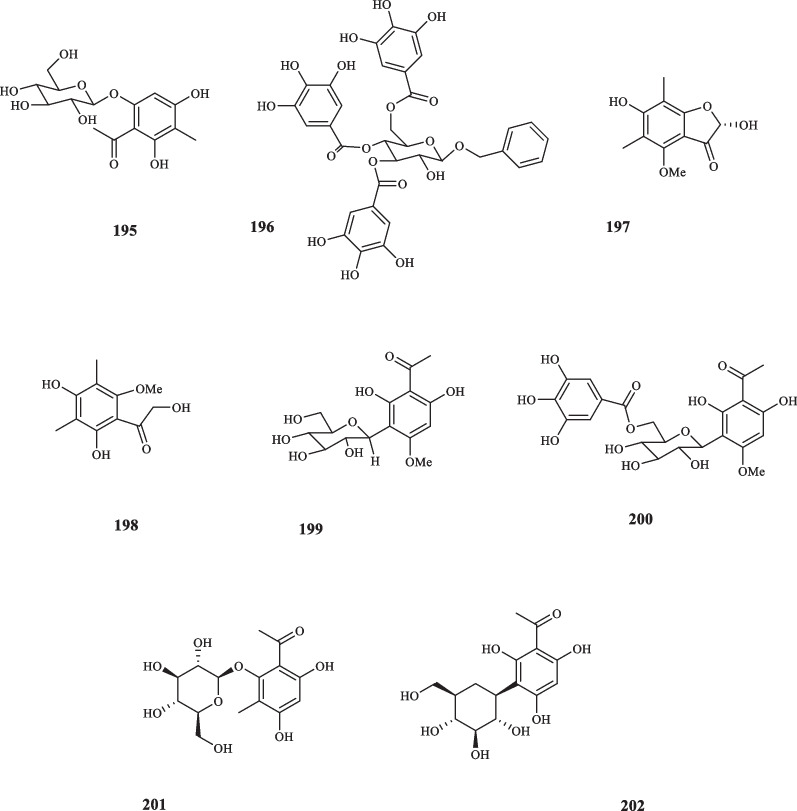
Acetophenone derivatives reported from the family Myrtaceae

Family Myrtaceae consists of about 142 genera and 5500 species. Myrtaceae is well-defined for its glandular leaves that contain aromatic polyphenolic and terpenoid substances [101, 102]. The majority of Myrtaceae species are trees that mainly distributed in tropical to temperate regions [102]. The EO of Mytraceae plants is primarily consisted of monoterpenes and sesquiterpenes, and often the mixture of both. Complex terpenes, such as triterpenes are also found in less values. Other compounds, like alkyl derivatives, β-triketones and aromatic compounds, such as acetophenone compounds also occur in the species, however, less commonly [102]. *Eucalyptus gomphocephala* is one of the most widely used plants with versatile uses across the globe and it has been used for therapeutic purposes since ancient times for its antiseptics and respiratory tract infection inhibitory features, etc. [103]. An experiment, designed for identifying the phenolic compounds from the *E. gomphocephala*, afforded two novel acetophenone derivatives, namely 2,4,6-trihydroxy-5-methyl-acetophenone 2-*O*-β-D-glucopyranoside (**195**) and Benzyl alcohol 7-O-(3',4',6'-tri-*O*-galloyl)-β-D glucopyranoside (**196**) [103]. **195** exhibited no cytotoxic activity on HeLa cells, the trait which **196** possessed at extremely low levels with an IC_50_ value of 367.1 *µ*M [103]. Ha et al. isolated two acetophenone compounds from the foliage of *Cleistocalyx operculatus* (**197** and **198**) [104]. Both compounds revealed to be moderately effective against neuraminidase of various strains of swine influenza virus including H9N2, H1N1, H1N1 (WT), and H274Y. **197** expressed inhibitory effects at the IC_50_ values of 38.00, 45.57, 43.84, and 36.72 *µ*M, respectively, as did **198** at the IC_50_ values of 40.33, 48.25, 44.13, and 48.07 *µ*M in the same order [104]. The screening of the extract from the cloves of *Syzygium aromaticum* revealed two previously-unknown acetophenones (**199** and **200**) [105]. Both compounds were subjected for their inhibitory abilities of prolyl endopeptidase, the result of which demonstrated the IC_50_ values of 218.9 and 17.2 *µ*M attributed to **199** and **200,** respectively. This assay shed light on the potent applicability of **199** for preventing memory loss [105]. Ryu et al. also isolated phloroacetophenone-*O*-glycoside compounds, 2,4,6-trihydroxy-3-methylacetophenone-2-*O*-β-D-glucoside (**201**) and 2,4,6-trihydroxyacetophenone-3-C-β-D-glucoside (**202**) from *S. aromaticum*; **201** was obtained from the flower buds of the plant [106], and **202** from both flower buds and its leaves [106, 107]. The cytotoxic activities of **201** and **202** were assessed against A2780 human cancer cells which disproved the pertaining abilities of both compounds as they both showed the inhibitory effect with the IC_50_ of > 100 *µ*M [106]. These novel acetophenone compounds are shown in Fig. 9.

### Other Families

Four acetophenone derivatives (**203–206**) were extracted from *Iris japonica* (Iridaceae) by Shi et al. [108]. Hoang et al., also, identified apocynin acetophenone (**207**) from *Iris spp* [109]. Apocynin (**207**) is an important naturally occurring acetophenone with various pharmacological activities. It has been studied for its potential in treating a variety of disorders, including diabetic complications, neurodegeneration, cardiovascular disorders, lung cancer, hepatocellular cancer, pancreatic cancer, and pheochromocytoma [110–113]. It has been formulated into various nanoparticles to enhance its absorption and duration of action [114]. Additionally, apocynin has shown promise in the treatment of various disorders, including diabetic complications, neurodegeneration, cardiovascular disorders, lung cancer, hepatocellular cancer, pancreatic cancer, and pheochromocytoma [112]. Its primary reported mechanism of action is as an NADPH oxidase (NOX) inhibitor, but recent studies have also highlighted its off-target effects, such as scavenging non-radical oxidant species [115]. Zulfiqar et al. isolated three acetophenone C-glycosides (**208**–**210**) from the stem of *Upuna borneensis* (Dipterocarpaceae) using acetone solvent [116]. Lendl et al. screened the extract of *Chione venosa (*sw*)* (Rubicaceae*)* and identified three acetophenone derivatives as: *ortho*-hydroxy-acetophenone-azine (**211**), acetophenone-2-*O*-[β-D-apiofuranosyl-(1″ → 6’)-*O*-β-D-glucopyranoside] (**212**), and acetophenone-2-*O*-β-D-glucopyranoside (**213**) [117]. A polyoxygenated acetophenone, denoted as 2,6-dimethoxy-4-hydroxyacetophenone (**214**) was identified in the bulbs of *Pancratium maritimum* (Amaryllidaceae) [118]. Analyzing the constituents of *P. biflorum* led to the identification of two previously-undiscovered acetophenone glycosides, named 4,6-Dimethoxyacetophenone-2-*O*-*β*-D-glucoside (**215)** and 2,6-Dimethoxyacetophenone-4-* O*-*β* -D-glucoside (**216**) [119]. Miyazawa and Kawata detected the presence of two acetophenone compounds, namely paeonol (**217**) and acetanisole (**218**), in the essential oil of *Cimicifuga simplex* (Ranunculaceae) [120]. Paeonol (**217**), a significant bioactive acetophenone, shows a range of pharmacological and biological activities. It has been shown to possess substantial anticancer effects, including the induction of apoptosis, inhibition of cell proliferation, and modulation of multiple signaling pathways [121]. Paeonol also shows potential as a therapeutic agent for atherosclerosis, with anti-atherosclerotic effects and protective effects on important cell types involved in the disease [122]. In osteoarthritis, paeonol has been found to mitigate inflammation, prevent extracellular matrix degradation, and inhibit chondrocyte apoptosis through the activation of the SIRT1 pathway [123]. Paeonol has proven to be effective in treating chronic dermatitis, reducing scratching behavior and skin inflammation [124]; It also has therapeutic effects in ulcerative colitis (UC) [125]. It has also shown promise in treating dry skin diseases by reducing inflammation and itching behavior through the CXCR3 pathway [124]. Furthermore, paeonol has various applications in different industries. It has been found to effectively inhibit the growth of Aspergillus flavus, a fungus that can damage agricultural products [126]. Paeonol treatment can also promote reendothelialization, the process of regrowing the endothelial layer of blood vessels, which is important for the treatment of vascular diseases [127].

Sancin (1971) identified acetophenone derivative, named acetovanillone (**219**) in the roots of *Apocynum venetum* (Apocynaceae) [128]. It also yielded *p*-hydroxyacetophenone (**126**) that was hitherto unidentified in the plant [128]. Research into *Polygonum multiflorum* from Polygonaceae family afforded a novel acetophenone compound, called polygoacetophenoside (**220**) [129]. Screening the chemical composition of *Rumex aquatica* extract, Yoon et al. extracted a new acetophenone compound, named rumexin (**221**) [130].

The roots of *Sanguisorba minor* from Rosaceae family produced 2′,6′-dihydroxy-4′-methoxyacetophenone (**222**) [131]. **222** exerted its phytoalexin characteristics by inhibiting the germination of two strains of fungi, namely *Botrytis cinerea* and *Phomopsis perniciosa* with the ED_50_ values of 45 and 410 *µ*M, respectively [131]. Prasad (1998), exploring the therapeutic constituents of *Prunus armeniaca*, extrcated the aromatic glycoside acetophenone, named 4-*O*-glycosyloxy-2-hydroxy-6-methoxyacetophenone (**223**) [132]. Acetophenone derivatives, knema pachycarpa A (**224**), and knema pachycarpa B (**225**) were isolated from the stems of *Knema pachycarpa* (Myristicaceae) [133]. Both compounds were evaluated for their cytotoxic activities against three cancer cell lines, including Hela, MCF7, and Hep3B; while both **224** and **225** reflected moderate activities against Hela cells with the IC_50_ values of 26.92 and 30.20 *µ*M, neither of them performed noticeable inhibitory activities against MCF7 and Hep3B cell lines (52.88 and 46.22 *µ*M against the former, and > 100 and 70.80 against the latter, respectively) [133].

Kanchanapoom et al. detected two acetophenone compounds from E*rythroxylum cambodianum* (Erythroxylaceae) (**226** and **227**) [134]. Acetophenone glycoside **(228)** was isolated from *Cassia sophera* (Fabaceae) [135]. Edayadulla and Ramesh pinpointed the occurrence of a novel prenylated acetophenone compound, 1-[2,4-dihydroxy-5-(3-methylbut-2-enyl)phenyl]ethenone (**229)** a derivative of 2,4-dihydroxyacetophenone, from *Derris indica* (Leguminosae) [136]. Kuang et al. extracted 3,5-dimethyl-6-hydroxy-2-methoxy-4-*O*-D-glucopyranosyl-oxy-acetophenone (**230**) from *Dryopteris fragrans* (Asplenaceae) [137]. Aladesanmi et al. isolated *p*-hydroxyacetophenone (**127**) for the first time from the leaves of *Dysoxylum lenticellare* (Meliaceae) [138]. In addition, Wei-sheng et al. isolated the same compound from *Saxifraga stolonifera* (Saxifragaceae) that was previously unknown in the genus [139]. The research into the identification of acetophenone components from different species edged into sampling herbal products, as Quispe et al. extracted also **127** and its glucoside derivatives from an infusion made of the aerial parts of *Fabiana imbricata* (Solanaceae) [140]. An acetophenone glycoside was isolated from *Exacum affine* (Gentianaceae) by Kuwajima et al. who defined it as affinoside (**231**) [141]. 2,3,4-Trihydroxy-5-methylacetophenone (**232**) was extracted from the sap of *Borassus flabellifer Linn* (Arecaceae) [142]. The antibacterial activity of **232** was put to test and it inhibited the growth of seven strains of bacteria, namely *B. cereus*, *E. coli*, *K. pneumoniae*, *M. smegmatis*, *S. aureus*, *S. epidermidis*, and *S. simulans,* with the MIC values of 62.5, 62.5, 125, 125, 62.5, 250, and 250 μg/mL, respectively. Regarding the anti-oxidant activity, compound **232** displayed an IC_50_ value of 20.02 *µ*M for the DPPH radical scavenging activity [142]. Cortex Moutan, the root bark of *Paeonia suffruticosa* (Paeoniaceae), was revealed to contain paeonol acetophenone (**217**) [143]. In vitro examination identified this compound as the anticoagulative agent of the plant, a quality which was posited to stem from the compounds involvement in the formation of A2 from arachidonic acid [143]. Three decades later, another member of Paeoniaceae family, *Paeonia ostia*, was found to host two novel acetophenones, namely 2-hydroxy-4-methoxy-acetophenone-3-*O*-[β-D-apiofuranosyl (1 → 6)-β-Dglucoside (**233**), and 4-hydroxy-2-*O*-β-rutinosyl acetophenone (**234**) [144]. Neither of these compounds possessed any significant anti-inflammatory activity as they failed to inhibit nitric oxide production in vito.

**Fig. 10:**
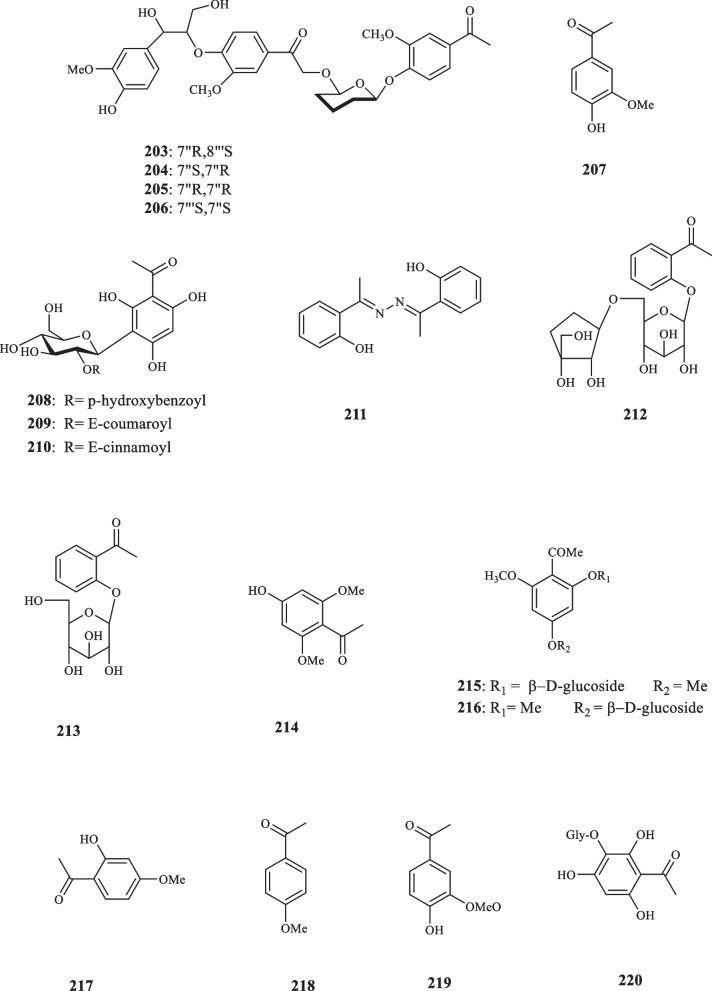

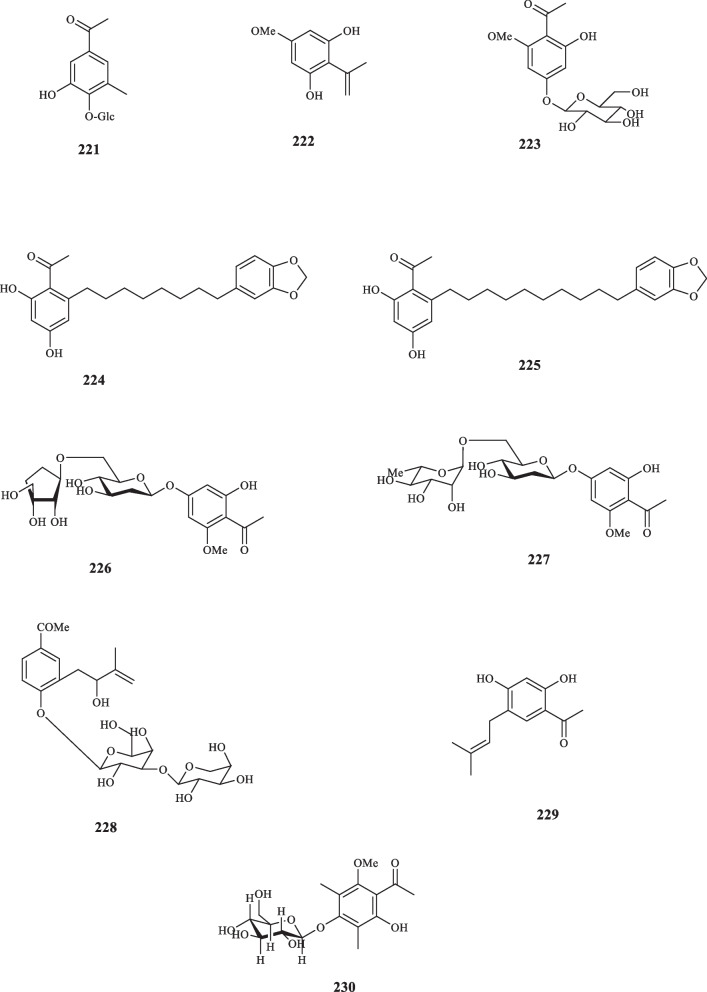

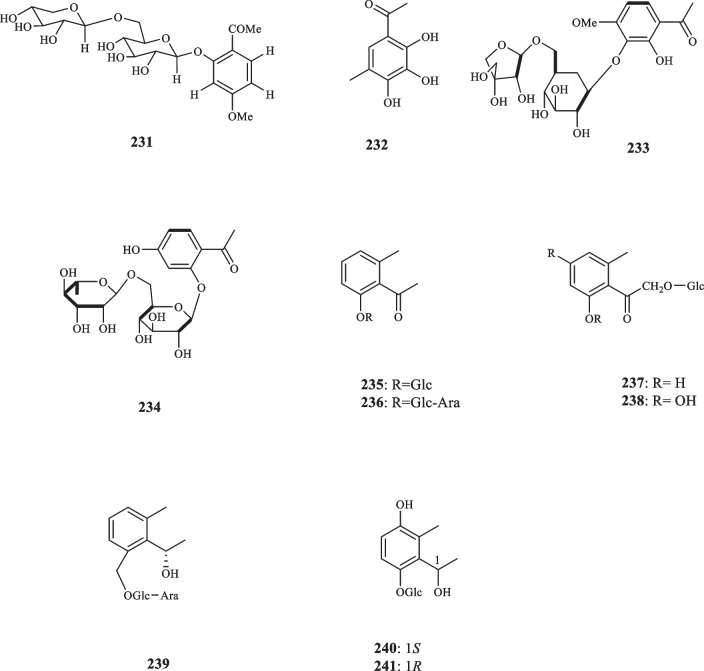
Acetophenone derivatives reported from the other families

There are reports of the presence of acetophenone compounds in Gymnosperms. Osswald et al. reported the extraction of *p*-hydroxyacetophenone (**127**) in *Picea abies* (spruce needles) (Pinaceae) and explored the fungitoxic activity of that compound towards *Cladosporium cucumerinum* and *Ospbaera kalkhoffii* [145]. Attempts to screen the compounds from the members of gymnosperms perpetuated as Inatomi et al. identified seven acetophenone derivatives from *Juniperus occidentalis* (Cupressaceae). These compounds were elucidated as Juniperoside III – IX (**235**–**241)** [146]. Figure 10 illustrates the aforementioned acetophenone compounds.

## Acetophenone derivatives produced by fungi

**Fig. 11:**
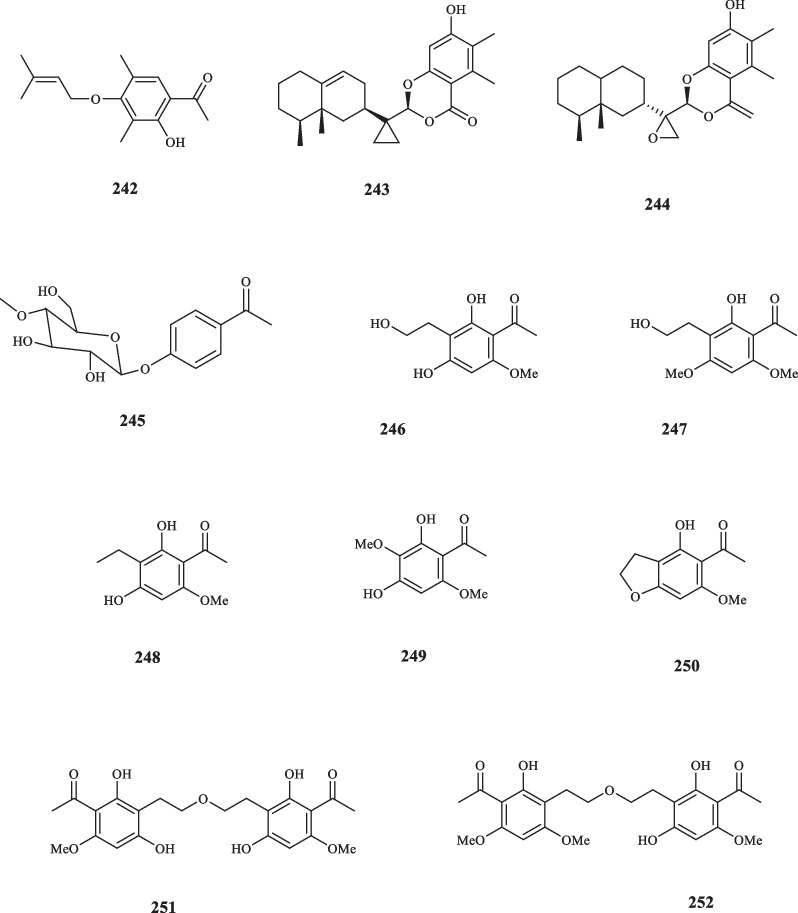
Fungi-derived acetophenone compounds

Fungi are a diverse group of organisms, with over 5 million estimated species [147], However, only a small fraction of these species have been cultivated, described, and studied for their chemical properties [148]. They have recently garnered significant attention from the scientific community due to their ability to produce bioactive secondary metabolites with novel structures and there have been attempts to isolate acetophenone compounds from various strains of fungi [149]. A novel acetophenone derivative, identified as 4-prenyloxyclavatol (**242**) was identified in and extracted from *Nigrospora sphaerica* fungus [150]. Eremophylane acetophenone conjugates, colletotricholides A (**243**) and colletotricholides B (**244**) were extracted from *Colletotrichum gloeosporioides* XL1200, an endophytic fungus collected from *Salvia miltiorrhiza* [151]. Compounds **243** and **244** showed no inhibitory effects towards any strains of pathogenic microorganism tested i.e., bacteria and fungi [151]. Another strain of fungi, referred to as *Mycosphaerella* sp. L3A1, was recently discovered to contain a previously undetected acetophenone, namely acetophenone-4-*O*-methyl-β-D-glucopyranoside (**245**). This compound proved ineffective against a group of cancer cells (MDA-MB-231, MDA-MB-435, HCT116, SNB19, PC-3, and A549) [152]. *Lindgomyces madisonensis* (G416) represents another fungal strain that yielded seven novel acetophenone compounds upon being isolated from submerged wood. These compounds were extracted and elucidated as madisone (**246**), 4′-demethoxydimadisone (**247**), dehydromadisone (**248**), 2″-methoxymadisone (**249**), dihydroallovisnaginone (**250**), dimadisone (**251**), and 4′-methoxydimadisone (**252**). Fungi-derived acetophenone compounds are elucidated in Fig. 11.

A summary of the biological activities of acetophenones are listed in Table 1.

**Table 1:** Biological activities reported from isolated acetophenones in detail

Compound	Species	Inhibition	Inhibitory value		References
Anti-inflammatory					
(**1**)	*Acronychia oligophlebi*	RAW 264.7 cells	IC_50_ (µg/mL)	26.4	Chen et al. [37]
(**3**)				46.0	
(**4**)				79.4	
(**5**)				57.3	
(**72**)	*Melicope pteleifolia*	Human PBML 5-LOX		0.42	Shaari et al. [21]
(**90**)	*Melicope semecarpifolia*	fMLP/CB-induced superoxide anion		21.37	Chen et al. [14]
		fMLP/CB-induced elastate release		27.35	
(**91**)		fMLP/CB-induced superoxide anion		23.24	
		fMLP/CB-induced elastate release		26.62	
(**92**)		fMLP/CB-induced superoxide anion		30.61	
		fMLP/CB-induced elastate release		28.73	
Cytotoxicity					
(**8**)	*Acronychia oligophlebia*	MCF-7	IC_50_ (µg/mL)	56.8	Niu et al. [16]
(**9**)				40.4	
(**10**)				69.1	
(**12**)				33.5	Yang et al. [38]
(**13**)				80.2	
(**14**)				71.1	
(**15**)				46.3	
(**16**)				25.6	
(**17**)	*Acronychia pedunculata*	Human KB tissue culture	IC_50_ (µg/mL)	0.5	Wu et al. [39]
		A-549		0.98	
		L-1210		2.95	
		P-388		3.28	
(**33**)		P-388	IC_50_ (µg/mL)	15.42	Tanjung et al. [45]
(**34**)		HT29		21.8	Panyasawat et al. [46]
		HeLa		14.2	
(**35**)		HT29		21.8	
		HeLa		14.2	
(**37**)	*Acronychia trifoliolata*	A549		26.6	Miyake et al. [48]
		KB		25.6	
		KB-VIN		19.2	
		MDA-MB 231		> 40	
		MCF-7		30.8	
(**38**)		A549		19.9	
		KB		20.4	
		KB-VIN		16.2	
		MDA-MB 231		22.6	
		MCF-7		19.4	
(**88**)	*Melicope quercifolia*	HeLa		2.6	Saputri et al. [28]
(**99**)	*Melicope barbigera*	A2780 cells		30	Le et al. [31]
(**126**)	*Eupatorium fortune*	MCF-7 cells		82.15	Chang et al. [59]
		A549 cells		86.63	
(**127**)		MCF-7 cells		> 100	
		A549 cells		> 100	
(**166**)	*Cynanchum taiwanianum*	PLC/ PRF/5	ED_50_ (µg/mL)	6.6	Huang rt al. [79]
		T-24		3.5	
(**186**)	*Euphorbia fischeriana*	AGS cancer cells	IC_50_ (*µM*)	39.85	Du et al. [88]
		Hep-G2 cancer cells		35.06	
(**193**)	*Euphorbia ebracteolate*	Hela-60 cancer cells		0.095	Zhang et al. [92]
		MCF-7 cancer cells		6.85	
		A-549 cancer cells		8.71	
		SMMC-7541 cancer cells		16.52	
(**194**)		Hela-60 cancer cells	IC_50_ (μg/mL)	2.69	
		MCF-7 cancer cells		0.346	
		A-549 cancer cells		0.879	
		SMMC-7541 cancer cells		12.86	
(**195**)	*Euphorbia fischeriana*	Hep-G2	IC_50_ (*µ*M)	33.9	Du et al. [88]
		Hep-3B		30.2	
		A549		39.4	
		NCI–H460		27.3	
		AGS		44.8	
(**196**)		Hep-G2		27.8	
		Hep-3B		25.4	
		A549		37.2	
		NCI–H460		31.6	
		AGS		38.2	
(**197**)		Hep-G2		45.4	
		Hep-3B		> 50	
		A549		37.2	
		NCI–H460		36.4	
		AGS		> 50	
(**200**)	*Mallotus japonicus*	HeLa cells	ID_50_ (µg/mL)	ID_50_ 14.80	Arisawa et al. [97]
(**201**)				0.28	
(**202**)				49.10	
(**203**)				8.80	
(**204**)	*Mallotus japonicus*	Leukemia L-5178Y cells	ED_50_ (µg/mL)	6.10	Arisawa et al. [98]
		KB cells		2.40	
(**214**)	*Syzygium aromaticum*	prolyl endopeptidase	IC_50_ (*µ*M)	17.2	Han [74]
(**238**)	*Knema pachycarpa*	HeLa		26.92	Giap et al. [133]
		MCF7		52.88	
(**239**)		HeLa		30.20	
		MCF7		46.22	
		Hep3B		70.80	
Antibacterial					
(**45**)	*Euodia lunu-ankenda*	*Entercoccus faecium*	MIC_75_ (*µ*M)	2.6	Tran et al. [49]
		*S. aureus*		20.6	Tran et al. [49]
(**141**)	*Helichrvsum italicum*	*E. coli* B	MIC (µg/mL)	25	Tomás-Barberán et al. [66]
		*E. coli* K			
(**195**)	*Euphorbia fischeriana*	*E. coli P*		12.5	Du et al. [93]
		*Pseudomonas* *aeruginosa*		6.25	
		*S. aureus*		6.25	
		*B. subtilis*		6.25	
(**196**)		*E. coli P*		6.25	
		*Pseudomonas* *aeruginosa*		12.5	
		*S. aureus*		3.12	
		*B. subtilis*		3.12	
(**197**)		*E. coli P*		6.25	
		*Pseudomonas* *aeruginosa*		3.12	
		*S. aureus*		1.56	
		*B. subtilis*		3.12	
(**246**)	*Borassus flabellifer Linn*	*B. cereus*	IC_50_ (*µ*M)	62.5	Reshma et al. [142]
		*E. coli*			
		*S. aureus*			
Antiviral					
(**200**)	*Mallotus japonicus*	HSV-1	ED_50_ (µg/mL)	6.18	Arisawa et al. [97]
(**201**)				0.11	
(**202**)				48	
(**203**)				0.97	
(**210**)	*Cleistocalyx operculatu*	H9N2 strain	IC_50_ (*µ*M)	38.00	Ha et al. [104]
		H1N strain		45.57	
		H1N1 (WT) strain		43.84	
		H274Y strain		36.72	
(**211**)	*Cleistocalyx operculatu*	H9N2 strain		40.11	
		H1N strain		48.25	
		H1N1 (WT) strain		44.13	
		H274Y strain		48.07	
Antioxidant Activities					
(**190**)	*Euphorbia ebracteolate*	DPPH	IC_50_ (µg/mL)	34.62	Wang et al. [90]
(**246**)	*Borassus flabellifer Linn*	DPPH	IC_50_ (*µ*M)	20.2	Reshma et al. [142]
Antifungal activities					
(**86**)	*Melicope borbonica*	*Candida albicans*	MIA (*µ*g/mL)	25	Simonsen et al. [25]
		*Penicillium expansum*	MIA (*µ*g/mL)	15	
	*Citrus limon*	*Hendersonula toruloidea*	ED_50_ (mM)	0.8	Hartmann and Nienhaus [53]
		*Phytophthora citrophthora*			
(**87**)	*Melicope borbonica*	*Candida albicans*	MIA (*µ*g/mL)	> 50	Simonsen et al. [25]
		*Penicillium expansum*		20	
(**141**)	*Melicope borbonica*	*Cladosporium herbarum*	MIC (µg/mL)	10	Tomás-Barberán et al. [66]
		*Phyrhophthora capsica*		50	
		*Neurospora crassa*		100	
		*Penicillium italicurn*		100	
		*Penicillium digitalum*		50	
α-Glucosidase inhibitory activity					
(**100**)	Melicope *patulinervia*	α-glucosidase	IC_50_ (*µ*M)	41.68	Vu et al. [32]
(**101**)				6.02	
(**102**)				67.44	
Antihyperglycemic activity					
(**160**)	*Cynanchum wilfordii*	Hepatic gluconeogenesis	20 *µ*M ➝ 12.5% suppression		Jiang et al. [77]
			40 *µ*M ➝ 29.4% suppression		

## Conclusion

Natural products (NPs) are a diverse and abundant source of biologically active compounds with enormous potential for new drug discovery and other applications. In today's clinical landscape, more than half of all drugs in use are either natural products or their derivatives, with plants contributing no less than a quarter of this total [153]. NPs have been used for centuries to treat a wide range of diseases, and modern research has shown that they possess a wide range of biological activities, including cytotoxicity, antibacterial, antifungal, antiviral, and antiparasitic activity. NPs are produced by a variety of organisms, including microbes and plants [154]. However, only a small fraction of the world's biodiversity has been studied for its pharmaceutical potential, suggesting that the vast majority of NPs remain to be discovered. Acetophenone compounds which are present in a relatively wide variety of plant species and some strains of fungi are garnering increasing focus by natural products researches as they have proven to possess diverse biological activities. The exploration of various plant and fungal species has yielded 266 natural acetophenone compounds and derivatives, many of which exhibit a wide range of biological activities. This illustrates the depth of possibilities that the study of acetophenones can offer in the realms of medicine and science.

## Data Availability

Not applicable.
